# DHRS4-AS1 regulate gastric cancer apoptosis and cell proliferation by destabilizing DHX9 and inhibited the association between DHX9 and ILF3

**DOI:** 10.1186/s12935-023-03151-x

**Published:** 2023-12-01

**Authors:** Lei Xiao, Yang zhang, Qingqing Luo, Cao Guo, Zihua Chen, Chen Lai

**Affiliations:** 1 Department of General Surgery, Xiangya Hospital of Central South University, Xiangya Road No. 87, Kaifu District, Changsha, 410000 Hunan Province China; 2 Hunan Key Laboratory of Precise Diagnosis and Treatment of Gastrointestinal Tumors, Xiangya Hospital of Central South University, Changsha, 410000 Hunan Province China; 3 National Clinical Research Center for Geriatric Disorders, Xiangya Hospital, Central South University, Changsha, 410008 Hunan China; 4 Department of Oncology, Hunan Provincial People’s Hospital, The First Affiliated Hospital of Hunan Normal University, Changsha, 410000 Hunan Province China; 5 Key Laboratory for Molecular Radiation Oncology of Hunan Province, Xiangya Hospital, Central South University, Changsha, 410008 Hunan China

**Keywords:** DHRS4-AS1, Gastric cancer, DHX9, ILF3, NF-κB signaling pathway

## Abstract

Gastric cancer (GC) causes millions of cancer-related deaths due to anti-apoptosis and rapid proliferation. However, the molecular mechanisms underlying GC cell proliferation and anti-apoptosis remain unclear. The expression levels of DHRS4-AS1 in GC were analyzed based on GEO database and recruited GC patients in our institution. We found that DHRS4-AS1 was significantly downregulated in GC. The expression of DHRS4-AS1 in GC tissues showed a significant correlation with tumor size, advanced pathological stage, and vascular invasion. Moreover, DHRS4-AS1 levels in GC tissues were significantly associated with prognosis. DHRS4-AS1 markedly inhibited GC cell proliferation and promotes apoptosis in vitro and in vivo assays. Mechanically, We found that DHRS4-AS1 bound to pro-oncogenic DHX9 (DExH-box helicase 9) and recruit the E3 ligase MDM2 that contributed to DHX9 degradation. We also confirmed that DHRS4-AS1 inhibited DHX9-mediated cell proliferation and promotes apoptosis. Furthermore, we found DHX9 interact with ILF3 (Interleukin enhancer Binding Factor 3) and activate NF-kB Signaling in a ILF3-dependent Manner. Moreover, DHRS4-AS1 can also inhibit the association between DHX9 and ILF3 thereby interfered the activation of the signaling pathway. Our results reveal new insights into mechanisms underlying GC progression and indicate that LncRNA DHRS4-AS1 could be a future therapeutic target and a biomarker for GC diagnosis.

**Supplementary Information:**

The online version contains supplementary material available at 10.1186/s12935-023-03151-x.

## Background

Gastric cancer (GC) is the second leading cause of cancer-associated mortality worldwide [1–3]. Despite improvements in surgical techniques and targeted drug chemotherapy, the mortality rate of GC patients remains high. Furthermore, due rapid GC cell proliferation and strong anti-apoptotic ability, many GC patients are prone to tumor recurrence and drug resistance [4]. Therefore, explore the molecular mechanism of tumorigenesis and anti-apoptosis is essential to improve tumor treatment and patient prognosis.

Long noncoding RNAs (lncRNAs) are non-protein coding transcripts that are more than 200 nucleotides [5, 6]. Increasing evidence shows that lncRNAs play an important role in cancer progression. Specifically, lncRNAs regulate cancer cell death and proliferation has been verified in previous study [7]. For example, lncRNA LINC00473 promotes proliferation and inhibits cell apoptosis in non-small cell lung cancer cells [8]. Another lncRNA, HYPAL, promotes proliferation and is associated with poor prognosis in GC [9]. LncRNA HOXA11-AS promotes CG cell proliferation and invasion by forming scaffolds with PRC2, LSD1, and DNMT1 [10]. Several studies found that lncRNAs may function as scaffolds with miRNA or RNA binding protein (RBPs) [11, 12]. However, recent researches have proved that lncRNA may server as oncogene mediated regulation of tumor suppressor protein ubiquitination [13]. In this study, we report lncRNA DHRS4-AS1, a natural antisense transcript of *DHRS4* that is implicated in multiple cancers [14, 15]. LncRNA DHRS4-AS1 acts as a tumor suppressor in most cancers, including glioma, clear cell renal cell carcinoma, and neuroblastoma [16–18]. However, The expression and molecular mechanism of DHRS4-AS1 in gastric cancer have not been reported.

In current study, We found that lncRNA DHRS4-AS1 is significantly downregulated in GC tissues. We also observed that DHRS4-AS1 regulates GC proliferation and apoptosis in vitro and in vivo. Mechanistically, we demonstrate that DHRS4-AS1 simultaneously interacts with DHX9 and the E3 ligase MDM2, which destabilizes DHX9 expression by promoting MDM2-mediated ubiquitination. In addition, we verified that lncRNA DHRS4-AS1 could also inhibit the association between DHX9 and ILF3, Which was critical for activating NF-κB signaling pathway by DHX9. Our results reveal the molecular mechanisms of GC and provide a new therapeutic strategy.

## Materials and methods

### Bioinformatics analysis

GSE106815 and GSE65801 gene expression data were downloaded from the GEO dataset [19, 20]. We also performed lncRNA microarray analysis in 5 paired gastric cancer tissues of our institution to detect the lncRNA expression profile. We have already described the processing and analysis of data in the previous study [21].

### Clinical samples

The clinical GC tissue samples and adjacent normal tissues were obtained from the Gastrointestinal Department of the Xiangya Hospital, Central South University. There were 68 clinical GC tissue samples involved in this study and the patients got informed consent. All samples were collected within 30 min after resection and were stored at − 80 ℃ until use. All study procedures were approved by the Ethics Committee of Xiangya Hospital, Central South University.

### Cell culture and transfection

All GC cell lines (AGS, HGC-27, MGC-803, MKN45, and MMKN74 cells) and GES1 human gastric mucosal epithelial cells were purchased from the Cell Bank of Chinese Academy of Sciences (China, Shanghai). The cells were cultured and incubated in 1640 medium (Invitrogen, USA) containing 10% fetal bovine serum (Invitrogen, USA). HEK293 cell was purchased from Shanghai Cell Researcher Biotech Co., Ltd. The cells were cultured and incubated in DMEM containing 10% fetal bovine serum. The cells were cultured at 37 ℃ in a 5% CO_2_ incubator.

The GC cell were transfected with recombinant lentiviruses using Enhance P reagent purchased from Genechem company (Shanghai, China). according to the manufacturer’s protocol. GC cells were infected with lentivirus for 48 h and selected with 2 ug/mL puromycin for 2 weeks. The transfection efficiency was evaluated by qRT-PCR. In addition, the DHX9 and ILF3 Full-length or truncation were designed from Shanghai Cell Researcher Biotech Co., Ltd. Lipofectamine 2000 (Invitrogen, USA) was used for cell transfection. The transfection efficiency of DHX9 knockdown plasmid was detected through western blotting. The plasmid sequences are supplied in Supplementary Table 1.

### RNA isolation and qRT-PCR

Total RNA from cells and tissues was isolated using TRIzol reagent (Invitrogen, Carlsbad, USA). Reverse transcription was performed using a Reagent test kit (Yeasen, Shanghai, China). qRT-PCR was used to detect gene expression using SYBR Green Master Mix (Yeasen, Shanghai, China). The primers were designed by Servicebio (Wuhan, China). Primer sequences are listed in Supplementary Table 2.

### 5-Ethynyl-20-deoxyuridine (EdU) and cell counting kit-8 (CCK-8) assays

Cells were incubated with 50 mM EdU (RiboBio, Guangzhou, China) for 2 h. Nuclei were stained with Hoechst dye for 30 min. Images were taken from 5 different fields of view under a fluorescence microscope. The percentage of proliferating cells that released green fluorescence was calculated. Cell proliferation was measured using a CCK-8 assay kit (Yeasen, Shanghai, China). The cells were incubated with 10 µL CCK-8 reagent for 2 h. Absorbance at 450 nm was measured with a microplate reader.

### Colony formation assay

Almost 500 cells were plated in 6-well culture plates and incubated for 2 weeks. After washing with PBS, the cells were fixed with 4% paraformaldehyde for 20 min and stained with 1% crystal violet for 10–15 min. Images were captured using a microscope. Image J software was used to quantify the number of colonies.

### Flow cytometry and TUNEL assays

The slice were incubated with Proteinase K for 20 min with 20 ug/mL in room temperature. After washing by PSB and incubated with 50ul TdT Incubation buffer in the dark for 1 h (Yeasen, Shanghai, China). Then adding 1ug/mL PI solution in dark for 5 min. Apoptosis of the tissues was measured using a fluorescence microscope.

### Western blot and co-immunoprecipitation

Total protein from cells and tissues were isolated from samples homogenized in RIPA lysis buffer (Beyotime, Shanghai, China). The protein was separated by SDS–PAGE. Bands were transferred onto a polyvinylidene difluoride membrane and blocked with 5% skim milk for 1 h. The membrane was incubated with primary antibodies at 4 °C overnight. Washed the membrane with TBST buffer solution and incubated with secondary antibody for 1 h. Protein bands were visualized using ECL (Advansta, USA). The antibodies used in this study are listed in Supplementary Table 3.

For co-immunoprecipitation experiments, cell lysates were prepared using Cell Lysis Buffer for Western and IP (Beyotime, Shanghai, China). The lysates were incubated with 2–5 µg antibody at 4 °C overnight. Protein A/G Plus-Agarose was washed three times with buffer and was incubated with cell lysates at room temperature for 4 h. The lysates was centrifuged at 4 °C and 3000 rpm. Proteins were eluted into protein loading buffer and analyzed by western blotting.

### Nude mice xenograft experiments

The animal experiments were reviewed and approved by the Medical Experimental Animal Care Commission of Central South University. Adult nude male mice were maintained in a specific.

pathogen-free facility (12 weeks old) and purchased from Vital River (Beijing, China). The GC cells were stably transfected with lentivirus. The cells were subcutaneously injected below the foreleg on each side. The tumor growth monitored daily. The tumor volume (V) was calculated by V(mm^3^) = 0.5 × width^2^ × length.

### In situ hybridization and fluorescence in situ hybridization assays

Probes targeting DHRS4-SA1 were designed by Servicebio (Wuhan, China). Samples were digested with 20 µg/mL proteinase K at 37 °C for 30 min after deparaffinization and rehydration. Then, the samples were hybridized with 1 µM probe overnight. Afterward, the samples were washed with diluted SSC (2× SSC for 10 min at 37 °C, 1 SSC, 2× SSC for 5 min at 37 °C, and 0.5 SSC for 10 min at room temperature). The samples were incubated with blocking reagent at room temperature, followed by alkaline phosphatase anti-digoxin (anti-DIG-AP) for 30 min at room temperature. Finally, BCIP/NBT chromogen was added. The nuclei were counterstained with nuclear fast red solution. Images were captured from 5 different fields of view using a microscope. For FISH assays, the cells were fixed in 4% formaldehyde for 15 min, washed 2–3 times with phosphate-buffered saline (PBS), and treated with 20 µg/mL proteinase K for 5 min. The cells were then incubated with prehybridization solution at 37 °C for 1 h and were hybridized with 1 µM probe overnight at 37 °C. Next, the cells were washed with diluted SSC (2× SSC for 10 min at 37 °C, 1 SSC 2 × 5 min at 37 °C, and 0.5 SSC for 10 min at room temperature). The cells were treated with blocking reagent at room temperature and incubated with anti-DIG-AP for 30 min at room temperature. Finally, the cells were incubated with CY3-TSA. Nuclei were counterstained with DAPI. Images were captured using a fluorescence microscope.

### Biotinylated RNA pull-down assay

DHRS4-AS1 and DHRS41-AS1-antisense plasmids were cloned into PGEM-T vector containing a T7 promoter to prepare a plasmid template for in vitro RNA synthesis. This construct was linearized and transcribed with T7 RNA polymerase (MEGAscript T7 Transcript Kit, Thermo Fisher, USA). DHRS4-AS1 and DHRS41-AS1-antisense RNA were biotinylated in vitro and incubated with GC cell lysates. Then, magnetic beads were bound to labeled RNA with streptavidin and the bound proteins were recovered using elution buffer included in a Pierce Magnetic RNA-Protein Pull-Down Kit (Thermo Fisher, USA). The RNA/protein complexes were washed. The retrieved proteins were analyzed by liquid chromatography-tandem mass spectrometry (LC–MS/MS) or western blotting.

### RNA immunoprecipitation

RNA immunoprecipitation (RIP) assays were performed using an RIP kit (Gzscbio, Guangzhou, China). The cells were homogenized using the cell lysis buffer used for western blot and IP assays. Magnetic beads were pretreated with RIP buffer and incubated with 2–5 µg DHX9, MDM2, or IgG antibodies at 4 °C for 6–8 h. Then, this slurry was mixed with cell lysate and incubated at 4 °C overnight. The purified RNA was stored at − 80 °C or analyzed by qRT-PCR.

### Turnover assays

GC cells were incubated with 50 µg/mL CHX (Selleck, Shanghai, China) to block protein synthesis. Cells were collected after CHX treatment. Protein was isolated and analyzed by western blot.

### Immunofluorescence

GC cells were seeded on glass slides and fixed in 4% paraformaldehyde for 20 min. Then, the cells were washed three times with PBS and blocked with 1% bovine serum albumin for 30 min. The cells were incubated with DHX9 and MDM2 antibody at 4 °C overnight, washed with PBST, and incubated with secondary antibody of the corresponding species at room temperature in the dark for 1 h. Glycerol or neutral resin was used to mount and seal the slides. Samples were imaged with a fluorescence microscope.

### Statistical analysis

All experiments were performed with at least three biological replicates. Statistical analyses were performed using GraphPad Prism 8.0 and SPSS 25 software. The associations between DHRS4-AS1 and clinical pathology characteristics were analyzed by Chi-squared tests. DHRS4-AS1 expression and GC patient survival were tested using Kaplan–Meier plots and log-rank tests. Differences between two groups were analyzed using two-tailed unpaired Student’s *t* tests or paired Student’s *t* tests. *p* value of < 0.05 was used as the criterion of statistical significance.

## Results

### lncRNA DHRS4-AS1 is downregulated in gastric cancer

**Fig. 1:**
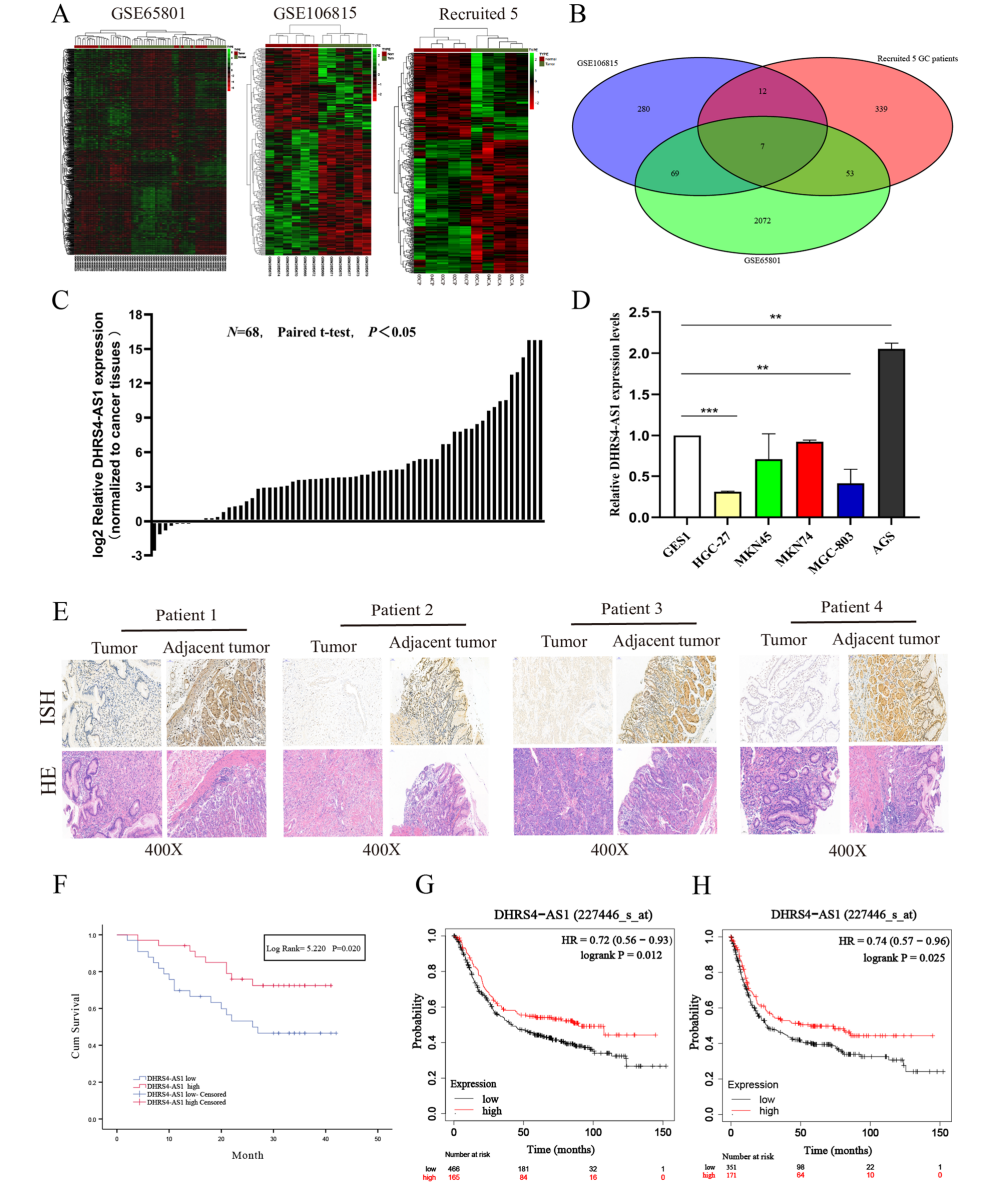
LncRNA DHRS4-AS1 is downregulated in gastric cancer. (**A**) Hierarchical clustering analysis of differentially expressed lncRNAs (fold change > 2; *P* < 0.05) in gastric cancer and normal tissues in gastric cancer and normal tissues. (**B**) overlap of misregulated lncRNAs in GEO datasets and our sequencing data. (**C**) Relative DHRS4-AS1 expression was analyzed by qRT-PCR in 68 GC tissue samples. (**D**) Relative DHRS4-AS1 levels in GC cell lines and normal human normal gastric mucosal epithelial cells. (**E**) LncRNA DHRS4-AS1 expression in GC tissue was analyzed by in situ hybridization. (**F**) The association between DHRS4-AS1 and OS was analyzed in our institution cohort. (**G** and **H**) Kaplan–Meier Survival Plots (K-M plots) analysis of the OS and PFS of GC patients based on DHRS4-AS1 expression. *p < 0.05, **p < 0.01, and ***p < 0.001

We First investigated the microarray datasets GSE65801 and GSE106815 to identified the misregulated lncRNA in GC tissues. There were 368 lncRNAs and 2201 lncRNAs misregulated in GC tissues with |logFC| > 2 and *P* < 0.05. In order to more accurately identify lncRNA closely related to gastric cancer, we recruited and collected 5 GC patients samples from our institution for lncRNA sequencing (Fig. 1A). We identified 7 misregulated lncRNAs common to all datasets (Fig. 1B). Among those 7 lncRNAs, DHRS4-AS1 was most remarkably down-regulated in these three datasets. To evaluate the function of DHRS4-AS1 in GC, the protein-coding potential of lncRNA DHRS4-AS1 was determined using the coding potential assessment tool (CPAT) [22]. The CPAT score for DHR4-AS1 was 0.259, which is blew 0.500 suggests that DHRS4-AS1 is a non-coding RNA (Fig. S1A). In addition, the ORF of lncRNA DHRS4-AS1 is negative value in txCdsPredict which created by University of California Santa Cruz(UCSC). It also indicated no protein-coding potential for DHRS4-AS1 (Fig. S1B).

We next examined DHRS4-AS1 expression using qRT-PCR in 68 paired GC tissues and adjacent normal tissues. LncRNA DHRS4-AS1 was significantly downregulated in GC tissues (Fig. 1C). To further investigate DHRS4-AS1 function, we examined DHRS4-AS1 expression in cell lines. Compared with human GES1 gastric mucosal epithelial cells, DHRS4-AS1 expression was significant downregulated except AGS cell line (Fig. 1D). In addition, ISH(In itu hybridization) assays revealed that DHRS4-AS1 was concentrated in normal gastric mucosal epithelial cells rather than tumor tissue (Fig. 1E). These data illustrate that lncRNA DHRS4-AS1 is tumor suppressor that might be involved in GC progression.

### DHRS4-AS1 expression is correlated with gastric cancer progression and poor prognosis

We divided patient samples into high and low DHRS4-AS1 expression groups according the median DHRS4-AS1 expression (Table 1). Low DHRS4-AS1 expression in GC tissues was significantly correlated with larger tumor size (*p* < 0.001), invasion depth (*p* = 0.021), TNM stage (*p* = 0.049) and vascular invasion (*p* = 0.028). We then used Kaplan–Meier (K–M) curves and log-rank method to analyze the correlation between DHRS4-AS1 enrichment and OS of GC patients. The result showed that the overall survival of the high DHRS4-AS1 group was significantly better than patients in the low DHRS4-AS1 group (*Log Rank* = 5.220 *p* = 0.020; Fig. 1F). Furthermore, the prognostic effect of DHRS4-AS1 was also supported by Kmplot database (www.kmplot.com) [23] (Fig. 1F and G). Furthermore, multivariate Cox proportional hazards analysis revealed that DHRS4-AS1 expression in GC was not an independent prognostic indicator (hazard ratio = 1.520; 95% CI 0.672–3.683; *p* = 0.353). Instead, the TNM stage was an independent prognostic indicator in GC (hazard ratio = 1.959; 95% CI 0.333–11.540; *P* = 0.046; Table 2). These data indicate that DHRS4-AS1 is a potential biomarker of poor prognosis in GC patients.

**Table 1:** The relationship of DHRS4-AS1 expression with clinicopathologic variable of Gastric Cancer patients(N = 68)

Clinicalpathology Characteristic	*n*	DHRS4-AS1		χ2	*P value*
		Low expression(n = 34)	High expression(n = 34)		
Age(years)					
<60	53	27	26	0.086	0.770
≥60	15	7	8		
Sex					
Male	44	24	20	1.030	0.310
Female	24	10	14		
Tumor size(cm)					
>5 cm	36	7	29	28.569	**<0.001** ^*^
≤ 5 cm	32	27	5		
Histologic differentiation					
Well and moderate	22	8	14	2.419	0.120
Poor and Undifferentiated	46	26	20		
Depth of invasion					
T_1_ + T_2_	11	2	9	5.314	**0.021** ^*^
T_3_ + T_4_	57	32	25		
Lymphatic metastasis					
Yes	28	12	16	0.971	0.324
No	40	22	18		
Metastasis					
M_0_	60	28	32	2.267	0.132
M_1_	8	6	2		
TNM stage					
I + II	28	10	18	3.886	**0.049** ^*^
III + IV	40	24	16		
Nerve invasion					
No	44	19	25	2.138	0.138
Yes	24	15	9		
Vascular invasion					
No	37	14	23	4.802	**0.028** ^*^
Yes	31	20	11		

**Table 2:** Univariate and multivariate Cox regression analyses DHRS4-AS1 for OS of patients in study cohort (n = 68)

Variables	OS		
	HR	95% CI	*P* value
Univariate analysis			
Age(≥ 60<60 vs. <60)	0.943	0.379–2.350	0.900
Sex(female vs. male)	0.867	0.393–1.912	0.724
Tumor size(≥ 5 cm vs<5 cm )	1.804	0.827–3.922	0.138
Histologic differentiation(poorly + undifferentiated vs. well + moderately)	3.273	1.127–9.507	**0.029** ^*^
Depth of invasion(T_3_ + T_4_ vs. T_1_ + T_2_)	6.667	0.902–49.273	0.063
Lymphatic metastasis(yes vs. no)	3.028	1.213–7.554	**0.018** ^*^
Metastasis(M_1_ vs. M_0_)	3.674	1.458–9.254	0.006
TNM stage(III + IV vs. I + II)	4.127	1.553–10.969	**0.004** ^*^
Nerve invasion(yes vs. no)	2.844	1.264−6.400	**0.012** ^*^
Vascular invasion(yes vs. no)	2.403	1.109–5.206	**0.026** ^*^
Expression of DHRS4-AS1 (high vs. low)	0.405	0.180–0.909	**0.028** ^*^
Multivariate analysis			
Lymphatic metastasis(yes vs. no)	1.380	0.286–6.670	0.688
Histologic differentiation(poorly + undifferentiatedvs well + moderately)	1.787	0.567–5.635	0.322
TNM stage(III + IV vs. I + II)	1.959	0.333–11.540	**0.046** ^*^
Vascular invasion(yes vs. no)	0.629	0.259–1.528	0.306
Expression of DHRS4-AS1 (low vs. high)	1.520	0.627–3.683	0.354

Abbreviations: CI, confidence interval; OS, overall survival; HR, hazard ratio. *P<0.05

### DHRS4-AS1 promotes gastric cancer cell apoptosis and inhibits proliferation in vitro

**Fig. 2:**
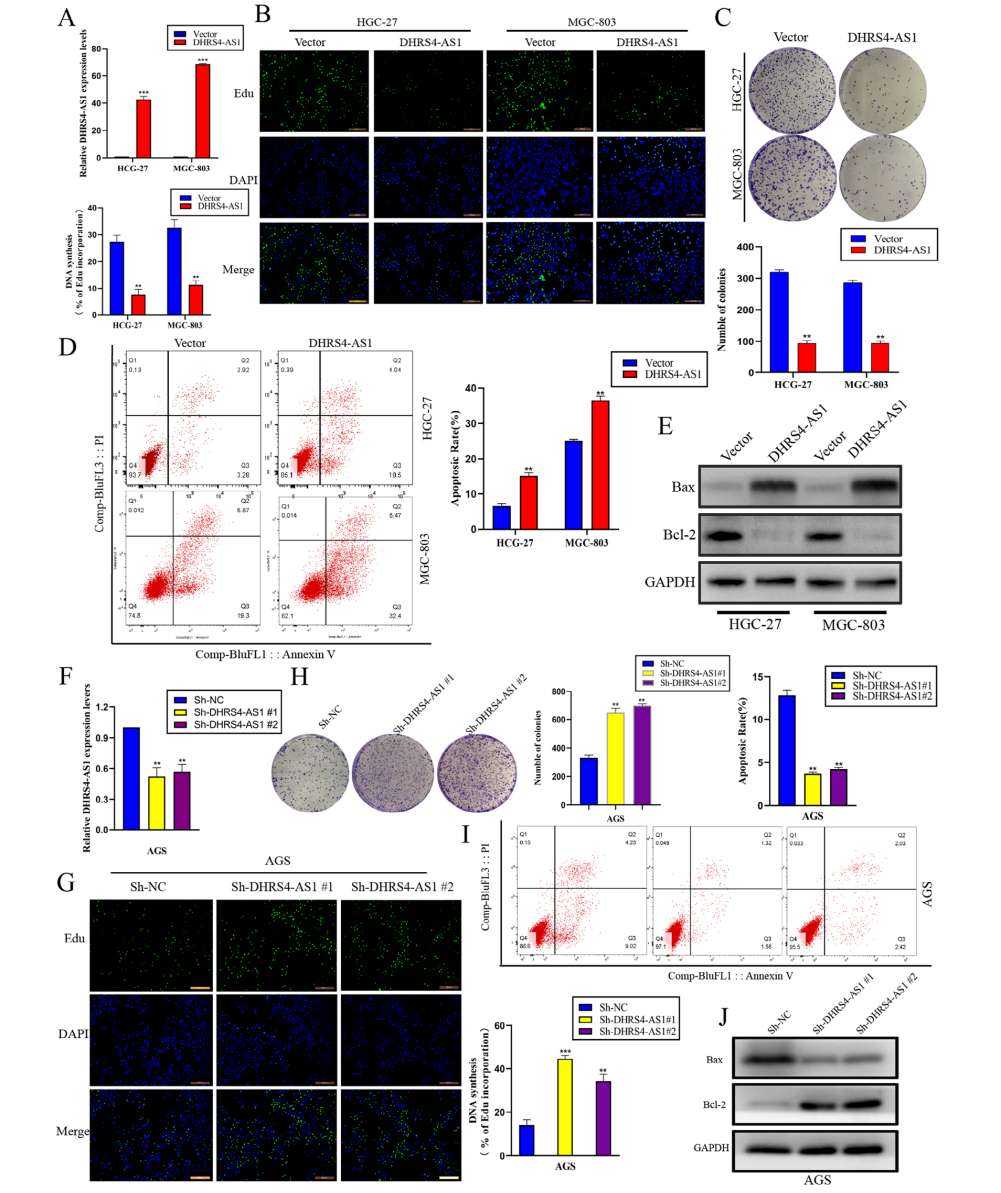
LncRNA DHRS4-AS1 promotes gastric cancer cell apoptosis and inhibits proliferation in vitro. (**A**) Relative DHRS4-AS1 expression was evaluated after transfection with DHRS4-AS1 Lentivirus. (**B** and **C**) EdU and clone formation assays to assess DHRS4-AS1-overexpressing GC cell proliferation. (**D**) Cell apoptosis was determined by flow cytometry after DHRS4-AS1 overexpressed. (**E**) Western blot assay of Bax and Bcl-2 expression in GC cells overexpressing DHRS4-AS1. (**F**) Relative DHRS4-AS1 expression was measured to evaluate transfection efficiency. (**G** and **H**) Clone formation assay and EdU assays were used to measure GC cell proliferation after transfection with DHRS4-AS1 knock-down plasmid. (**I**) Cell apoptosis was determined by flow cytometry after DHRS4-AS1 knockdown. (**J**) Western blot assays to evaluate Bax and Bcl-2 expression in GC cells after DHRS4-AS1 knockdown. *p < 0.05, **p < 0.01, and ***p < 0.001

To further investigate the function of DHRS4-AS1 in GC, we overexpressed lncRNA DHRS4-AS1 in HGC-27 and MGC-803 GC cells. These cells showed significant DHRS4-AS1 downregulation compared to GES1 cells (Fig. 1D). Transfection efficiency of DHRS4-AS1 overexpressed was detected by qRT-PCR (Fig. 2A). EdU assays demonstrated that DHRS4-AS1 significantly impaired cell proliferation (Fig. 2B). Further Colony formation assay results also showed that lncRNA DHRS4-AS1 inhibited colony formation in HGC-27 and MGC-803 cells (Fig. 2C). Furthermore, flow cytometry analysis was performed to investigate the effect of DHRS4-AS1 on GC cell apoptosis. We observed that DHRS4-AS1 overexpression significantly increased apoptosis in GC cells (Fig. 2D). We then performed western blot assays to examine the expression of the antiapoptotic protein Bcl-2 and the apoptosis indicator Bax. The results showed that DHRS4-AS1 overexpression significantly increased Bcl-2 expression and reduced Bax expression (Fig. 2E). Although, We downregulated DHRS4-AS1 expression in AGS cells, which had higher DHRS4-AS1 expression than GES1 cells. Transfection efficiency was investigated using qRT-PCR in AGS cells (Fig. 2F). EdU and colony formation assays revealed cell proliferation was significantly increased with lncRNA DHRS4-AS1 knockdown (Fig. 2G H). As expected, flow cytometry showed that GC cell apoptosis was decreased by sh-DHRS4-AS1 (Fig. 2I). Similarly, western blotting demonstrated that DHRS4-AS1 knockdown significantly upregulated the expression of the anti-apoptotic protein Bcl-2 and reduced Bax expression (Fig. 2J). Taken together, these results demonstrate that DHRS4-AS1 suppresses GC cell proliferation and promotes apoptosis in vitro.

### DHRS4-AS1 promotes gastric cancer cell apoptosis and inhibits proliferation in vivo

**Fig. 3:**
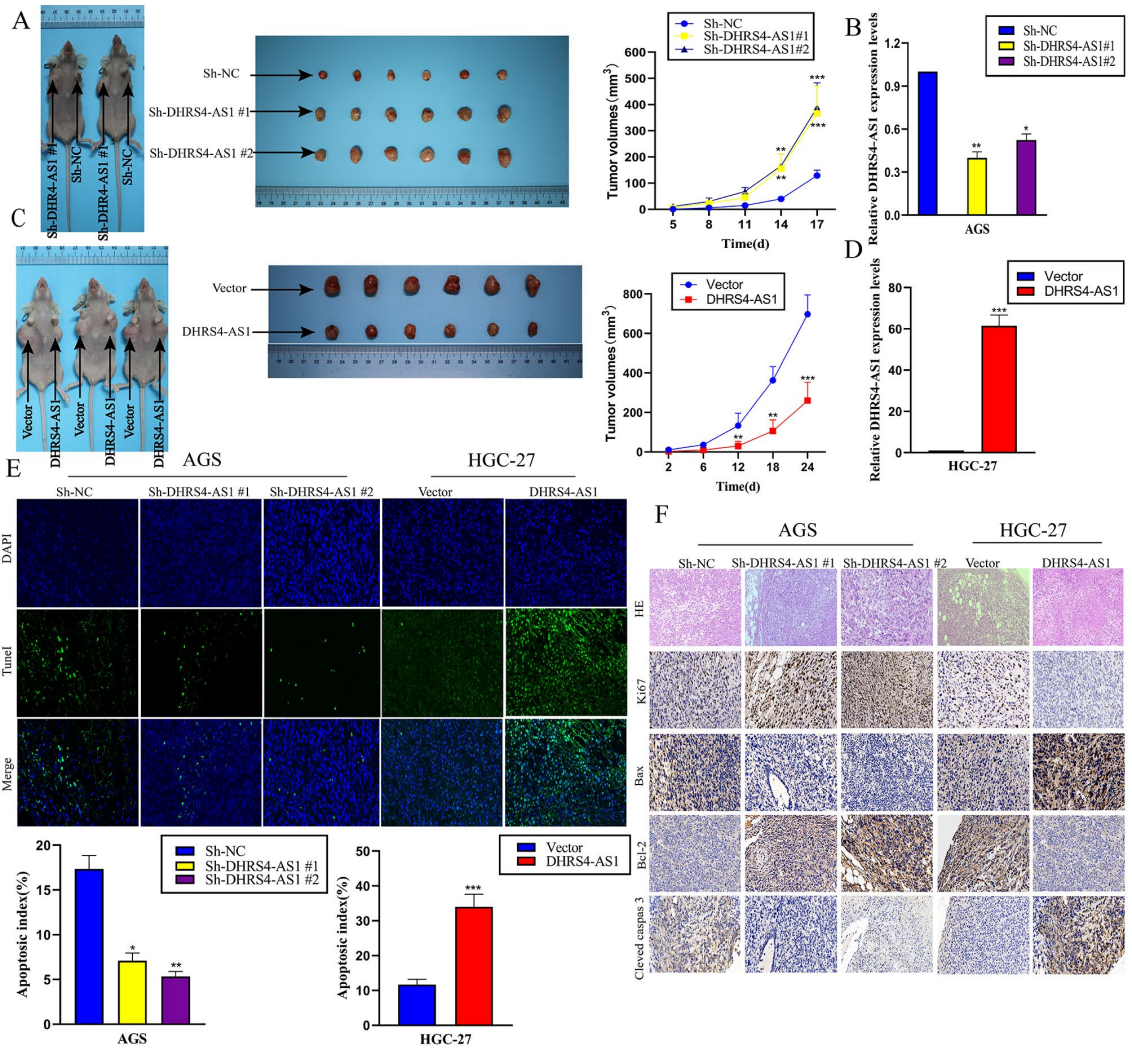
LncRNA DHRS4-AS1 promotes gastric cancer cell apoptosis and inhibits proliferation in vivo. (**A**) Tumor size in xenograft mice with tumors derived from Sh-NC-, Sh-DHRS4-AS1#1-, and Sh-DHRS4-AS1#2-expressing AGS cells was evaluated. Data represent the mean ± SD, n = 6. (**B**) Relative DHRS4-AS1 expression in mouse tumors was evaluated. (**C**) Vector- and DHRS4-AS1-overexpressing AGS cells were injected into mice. We evaluated tumor growth curves. Data represent the mean ± SD, n = 6. (**D**) Relative DHRS4-AS1 expression was evaluated in mouse tumors. (**E**) GC cell apoptosis was analyzed using TUNEL assays using samples from subcutaneously-implanted tumors with different DHRS4-AS1 expression. (**F**) Immunostaining of apoptosis-related proteins and Ki67 in subcutaneous tumors from DHRS4-AS1-expressing cells. *p < 0.05, **p < 0.01, and ***p < 0.001

We next injected DHRS4-AS1 stable knockdown AGS cells, DHRS4-AS1 overexpressing HGC-27 cells, or control cells into nude mice to investigate the effects of DHRS4-AS1 on GC cell tumorigenesis in vivo. Tumors derived from DHRS4-AS1 knockdown HGC-27 cells were larger than negative controls (Sh-NC) (Fig. 3A and B). Conversely, the tumors derived from DHRS4-AS1-overexpressing AGS cells were smaller than in the Vector group (Fig. 3C and D). IHC assays revealed that tumor tissues collected from the DHRS4-AS1 knockdown group had more Ki67-positive cells, whereas the DHRS4-AS1 overexpression group had fewer Ki67-positive cells than the Vector group (Fig. 3E). TUNEL assays were performed to assess tumor cell apoptosis. Apoptosis in AGS cells in the Sh-NC, Sh-DHRS4-AS1#1, and Sh-DHRS4-AS1#2 groups were 17.3 ± 0.23%, 7.1 ± 0.73% and 5.3 ± 0.87%, respectively. In contrast, apoptosis in HGC-27 cells from the Vector and DHRS4-AS1 groups were 11.7 ± 0.48% and 34.1 ± 0.93%, respectively (Fig. 3E). Moreover, higher Bax and cleaved caspase-3 levels were observed in the DHRS4-AS1 overexpression group compared with the Vector group. We also observed lower Bcl-2 levels in the DHRS4-AS1 overexpression group. Lower Bax levels and higher Bcl-2 and cleaved caspase-3 levels were observed in the Sh-DHRS4-AS1#1 and Sh-DHRS4-AS1#2 groups compared with the Sh-NC group in AGS cell-derived tumors. These results were similar to the in vitro results (Fig. 3F). Therefore, DHRS4-AS1 promotes GC cell apoptosis and inhibits proliferation in vivo.

### DHRS4-AS1 accelerates DHX9 degradation via the ubiquitin-proteasome pathway

**Fig. 4:**
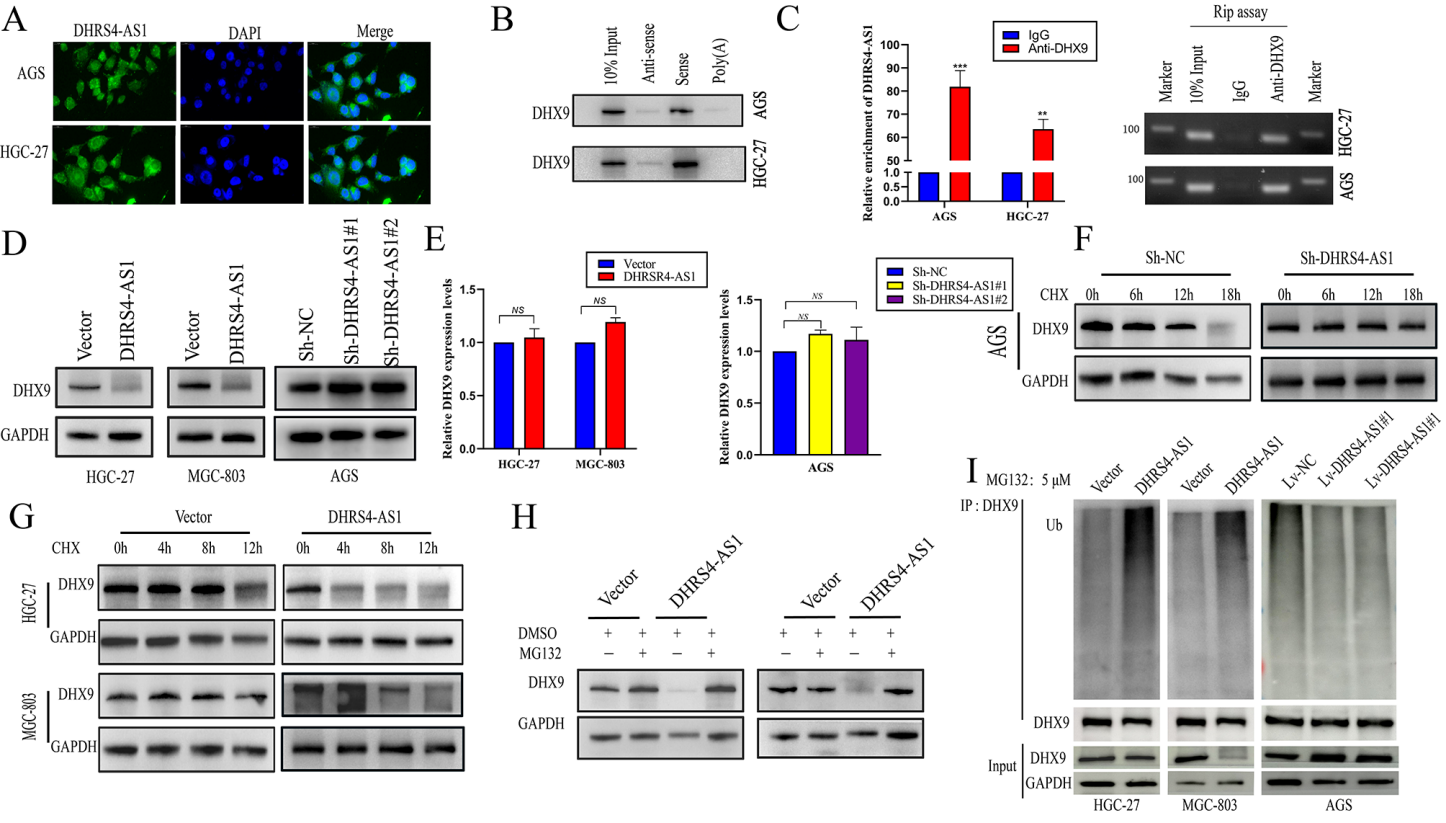
LncRNA DHRS4-AS1 binds DHX9 and accelerates its degradation. (**A**) FISH was used to detect DHX9 localization in GC, AGS, and HGC-27 cells. (**B**) LncRNA DHRS4-AS1 pull-down followed by western blotting to detect interactions with DHX9. (**C**) RIP assays were employed using DHX9 antibodies to determine if lncRNA DHRS4-AS1 co-precipitates with DHX9. IgG was used as a negative control that did not interact with DHRS4-AS1. (**D**) Western blot analysis showing that DHRS4-AS1 alters DHX9 expression. (**E**) qRT-PCR showed that DHRS4-AS1 has no effect on DHX9 mRNA levels. (**F**) DHX9 expression was measured in GC and AGS cells expressing Sh-DHRS4-AS1 or Sh-NC groups by western blotting after treatment with 50 µg/mL CHX. (**G**) DHX9 expression was measured in HGC-27 and MGD-803 cells overexpressing DHRS4-AS1 or empty vector using western blotting after treatment with 50 µg/mL CHX. (**H**) DHX9 expression in DHRS4-AS1-overexpressing and empty vector-expressing GC, HGC-27, and MGC-803 cells after treatment with 5 µM MG132 was evaluated using western blotting. (**I**) Western blot of DHX9 immunoprecipitated from DHRS4-AS-expressing GC cells to examine endogenous DHX9 ubiquitination.*p < 0.05, **p < 0.01,*NS*:Not significant

Accumulating evidence suggests that lncRNAs may function as scaffolds for binding proteins that promote cancer development [24–26]. To further investigate the mechanisms involving DHRS4-AS1 in GC cells. we first investigated the distribution of DHRS4-AS1 in AGS and HGC-27 cells using FISH assay. DHRS4-AS1 was distributed almost equally between the cytoplasm and the nucleus (Fig. 4A), which corroborates a previous study showing that almost 40% of lncRNA DHRS4-AS1 is distributed in the nucleus in HL7702 cells [12]. We next performed RNA pull-down assays with AGS nucleus extracts and identified proteins that interact with DHRS4-AS1 using mass spectrometry. We found that DHX9 was the fourth most abundant protein among all the pulled-down proteins (Supplementary Table 4). Western blotting using DHRS4-AS1 pull-down extracts from GC AGS and HGC-27 cells confirmed that DHX9 indeed binds to DHRS4-AS1 (Fig. 4B). Consistently, DHX9 antibody successfully pulled down lncRNA DHRS4-AS1 in RIP assays, which was confirmed by polymerase chain reaction (PCR) and quantitative PCR (qPCR) (Fig. 4C). Furthermore, DHX9 protein abundance was significantly decreased after DHRS4-AS1 overexpression, while DHRS4-AS1 knockdown upregulated DHX9 expression (Fig. 4D). However, qRT-PCR revealed that DHRS4-AS1 did not alter DHX9 mRNA levels (Fig. 4E), which indicates that DHRS4-AS1 does not reduce DHX9 protein expression by reducing DHX9 mRNA levels. We next examined DHX9 expression in AGS cells after transfection with Sh-NC, Sh-DHRS4-AS1#1, or Sh-DHRS4-AS1#2 after treatment with the protein synthesis inhibitor Cycloheximide(CHX). DHX9 expression in both DHRS4-AS1 knockdown groups decreased more slowly than in the Sh-NC group (Fig. 4F). Conversely, DHRS4-AS1 overexpression enhanced DHX9 downregulation after treating HGC-27 or MGC-803 cells with CHX (Fig. 4G). In addition, we examined whether lncRNA DHS4-AS1-mediated DHX9 downregulation is inhibited by the proteasome inhibitor MG132(5 µM). The results showed that MG132 could abolish down-regulation of DHX9 by lncRNA DHRS4-AS1 induced (Fig. 4H). We then examined whether DHX9 degradation was mediated by ubiquitination. Endogenous DHX9 was immunoprecipitated from GC cells, which showed that ubiquitin signals were increased in the DHRS4-AS1 overexpression groups compared with control cells. Consistently, DHX9 ubiquitination was decreased in cells in the Sh-DHRS4-AS1 group (Fig. 4I). These data indicate that DHRS4-AS1 regulates DHX9 expression via ubiquitin-proteasome system.

### DHRS4-AS1 promotes interactions between DHX9 and the E3 ligase MDM2

**Fig. 5:**
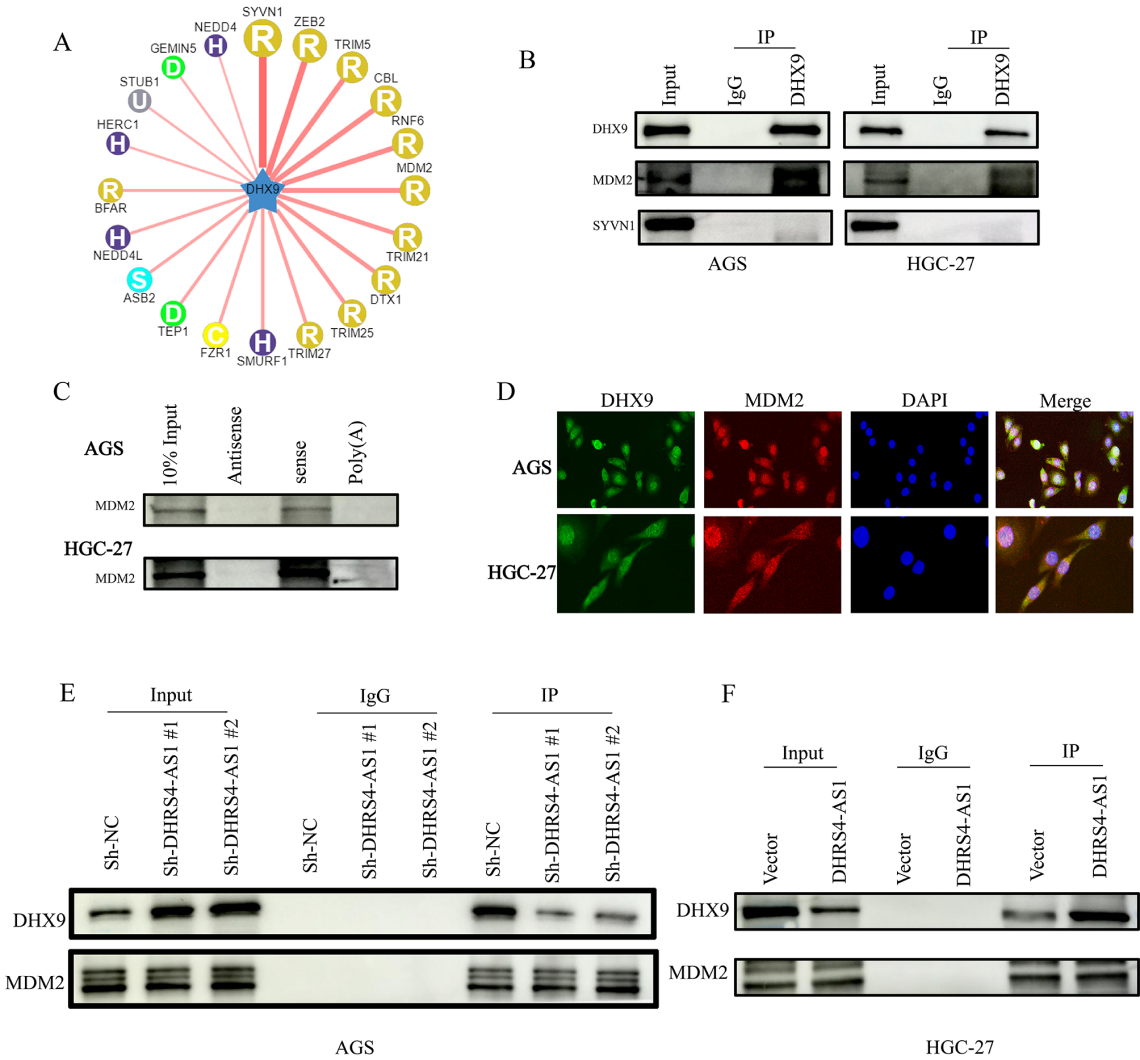
LncRNA DHRS4-AS1 promotes interactions between DHX9 and the E3 ligase MDM2. (**A**) Candidate E3 ligases that interact with DHX9 were predicted from the Human Protein Reference Database. (**B**) Immunoblotting following immunoprecipitation with an DHX9 antibody verified that MDM2, rather than SYVN1, interacts with DHX9. (**C**) DHRS4-AS1 pull-down followed by western blotting validated interactions with MDM2. (**D**) Immunostaining of DHX9 and MDM2 in AGS and HGC-27 cells. (**E**) Immunoblotting for DHX9 and MDM2 following immunoprecipitation from AGS cells from the Sh-NC, Sh-DHRS4-AS1#1, and Sh-DHRS4-AS1#2 groups. (**F**) Immunoblotting for DHX9 and MDM2 following immunoprecipitation in vector and DHRS4-AS1-overexpressing HGC-27 cells

We searched for candidate DHX9 ligases in the Human Protein Reference Database (www.hprd.org) to identify the potential ubiquitin E3 ligase that targets DHX9 in gastric cancer. The top 20 potential E3 ligases are shown (Fig. 5A and Supplementary Table 5). We next performed immunoprecipitation assays and found that the ubiquitin E3 ligase MDM2, rather than SYVN1, interacts with DHX9 (Fig. 5B). We also analyzed proteins pulled-down with DHRS4-AS1 and observed that MDM2 was pulled-down (Fig. 5C). In addition, MDM2 and DHX9 co-localized with each other in AGS and HGC-27 cells (Fig. 5D). We further examined whether lncRNA DHRS4-AS1 impacts interactions between DHX9 and MDM2 in GC cells. Immunoprecipitation assays showed that less DHX9 precipitated with MDM2 in AGS cells after DHRS4-AS1 knock down, compared with the Sh-NC group (Fig. 5E). In contrast, more DHX9 precipitated with MDM2 in HGC-27 cells after DHRS4-AS1 overexpressed (Fig. 5F). These results indicate that lncRNA DHRS4-AS1 acts as a scaffold to facilitate interactions between DHX9 and the E3 ligase MDM2, thus accelerating DHX9 degradation.

### DHX9 enhances cell proliferation and inhibits apoptosis in GC cells, which is impaired by lncRNA DHRS4-AS1

**Fig. 6:**
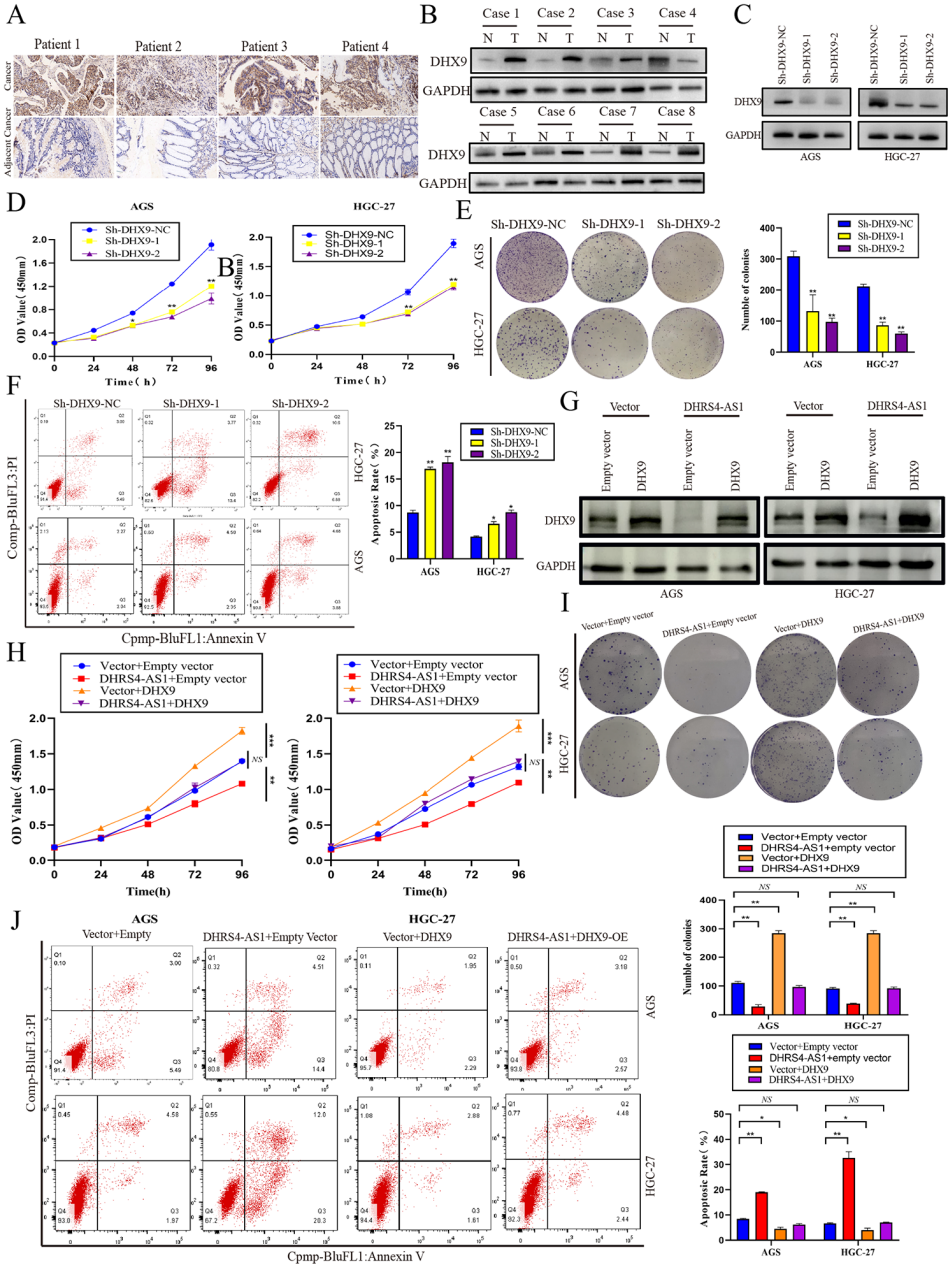
DHX9 is upregulated in GC and is inhibited by lncRNA DHRS4-AS1. (**A**) Immunohistochemistry was performed to detect DHX9 expression in gastric cancer and adjacent normal tissues. (**B**) Western blotting was used to detected DHX9 protein expression in gastric cancer and adjacent normal tissues. (**C**) Relative DHX9 expression in GC cells after transfection Sh-DHX9-NC, Sh-DHX9-1, and Sh-DHX9-2. (**D** and **E**) CCK-8 and clone formation assays to examine DHX9-transfected cell proliferation. (**F**) FCM of apoptosis in GC cells transfected with DHX9. (**G**) Relative DHX9 expression level was measured using western blotting in cells transfected with empty vector and DHRS4-AS1 overexpression groups. (**H**) CCK-8 assay measuring the proliferation of transfected cells. (**I**) Colony formation assay to examine cell proliferation after DHX9 knockdown. (**J**) FCM assay to examine apoptosis in transfected cells. *p < 0.05, **p < 0.01 NS:Not significant

DHX9 expression in GC was searched in TCGA and GEPIA databases. The results showed that DHX9 was significantly upregulated in GC tissues (Fig. S2A and S2B). To verify the TGCA results, we performed immunohistochemistry (IHC) to examine DHX9 expression in GC tissues. As expected, the IHC results showed that DHX9 expression in GC tissues was significantly higher than in paired adjacent normal tissue (Fig. 6A). Similar results showed that DHX9 protein level in GC tissues was significantly upregulated compared to adjacent cancer tissues (Fig. 6B). We next performed DHX9 knockdown in AGS and HGC-27 cells. The transfection efficiency was investigated using western blotting (Fig. 6C). CCK-8 assays and Clone formation assays showed that DHX9 knockdown impaired cell proliferation and clone formation (Fig. 6D and E). Moreover, flow cytometry revealed that DHX9 knockdown significantly increased GC cell apoptosis (Fig. 6F). These results illustrate that DHX9 is significantly upregulated and acts as an oncogene in GC. As we found both lncRNA and DHX9 regulate cell proliferation and apoptosis in GC. Moreover, lncRNA DHRS4-AS1 decreased DHX9 expression. Thus, we examined whether the effects of DHX9-mediated proliferation and apoptosis in GC cells were inhibited by lncRNA DHRS4-AS1. We transfected empty vector or DHX9-overexpression plasmids into the control and DHRS4-AS1 groups (Fig. 6G). DHRS4-AS1 decreased GC cell proliferation, while overexpressing DHX9 increased proliferation. However, lncRNA DHRS4-AS1 can impair DHX9-mediated proliferation.(Figure 6H and I) Besides, overexpressing DHRS4-AS1 increased GC cell apoptosis, while overexpressing DHX9 showed the opposite effect. However, lncRNA DHRS4-AS1 can impair DHX9-mediated apoptosis resistance (Fig. 6J). Our results indicate that DHX9 is upregulated in GC, and its effects on GC cell proliferation are inhibited by DHRS4-AS1.

### LncRNA DHRS4-AS1 inhibited association between DHX9 and ILF3 and inhibiting the activation of NF-κB signaling pathway by DHX9

**Fig. 7:**
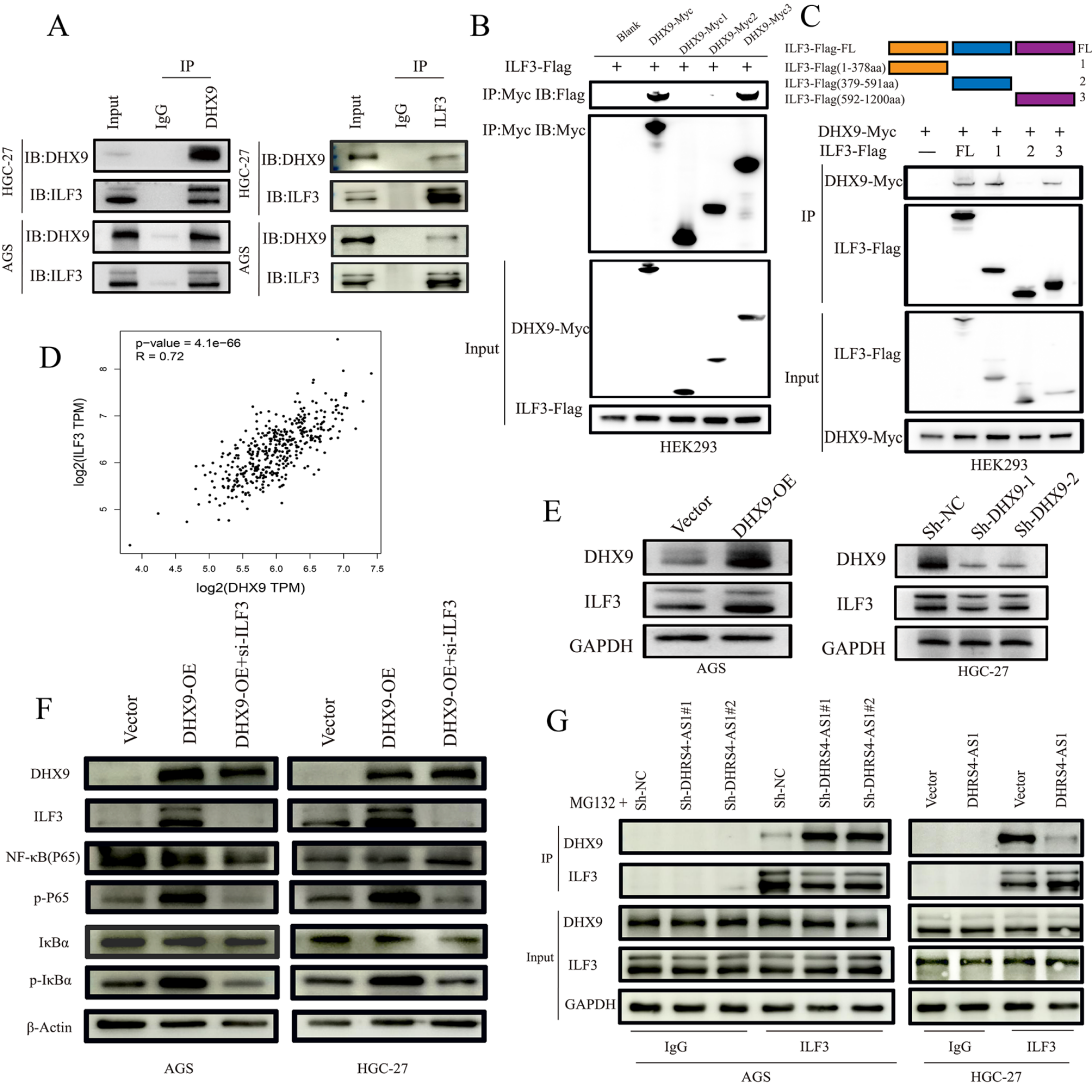
lncRNA DHRS4-AS1 inhibited the associate between DHX9 and ILF3. (**A**) Western blot analysis of co-IPs performed in AGS and HGC-27 with DHX9 and ILF3 antibody. (**B**) Western blot analysis of co-IPs performed on lysates prepared from HEK293 cells transfected with ILF3-Flag alone or together with indicated DHX9-Myc constructs. Top, co-IP performed with anti-Myc; bottom, input protein. (**C**) Western blot analysis of co-IPs performed on lysates prepared from HEK293 cells transfected with DHX9-Myc alone or together with indicated ILF3-Flag constructs. Top, CO-IP performed with anti-Myc; bottom, input protein. (**D**) The correlation between DHX9 and ILF3 was analysed in ChipBase V2.0 database. (**E**) Western blot analysis was performed to detected the expression of ILF3 in DHX9 overexpressd and knockdown GC cell. (**F**) Overexpression of DHX9 activate the phosphorylation of NF-κB (P65) and IκBα on protein level while was seriously alleviated by ILF3 knockdown. (**G**) Western blot analysis of co-IPs performed with anti-DHX9 and lysates prepared from DHRS4-AS1 overexpressd and knockdown GC cells. Cells were pretreated with MG132 (5 µM) for 90 min.IgG groups served as negative controls

To further reveal the molecular mechanism of DHX9 in gastric cancer, we conducted proteomic analysis using lysates prepared from DHRS4-AS1 pull-down. We found another cancer-related protein ILF3 was presented in DHRS4-AS1 pull-down complexes. We performed co-IPs in HGC-27 and AGS cells. Western blot analysis of CO-IP demonstrated that DHX9 and ILF3 associate with each other (Fig. 7A). To further map the regions of DHX9 and ILF3 responsible for their interaction. We performed Co-IP assay with ILF3-Flag and Myc-tagged truncated as well as full-length DHX9. Based on the signature protein domains: Myc 1, residues 1-254, Myc 2, residues 255–635, Myc 3, residues 636–1200. ILF3-Flag was only present in Co-IPs when the Myc 3 domains was present (Fig. 7B). It suggested Myc 3 domains of DHX9 were crucial for the interaction with ILF3. Besides, we also constructed Flag-tagged domains to test in coIPs with DHX9-Myc. Flag 1, residues 1-378, Flag 2, residues 379–591, Flag 3, residues 592–894. ILF3-Flag1 and ILF3-Flag3 pulled down DHX9-Myc at a similar efficiency as full-length (Fig. 7C). Those results indicated ILF3-Flag1 and ILF3-Flag3 were critical elements for complex formation between DHX9 and ILF3.

Furthermore, we found that DHX9 and ILF3 showed significant positive correlation in GC in ChIPBase V2.0 database (Fig. 7D). We also found ILF3 protein abundance was significantly decreased after DHX9 knockdown while DHX9 overexpression upregulated ILF3 expression (Fig. 7E). Therefore, we have reason to believe that ILF3 plays a crucial role in the function of DHX9. Previous study have proved that DHX9 contributes to the malignant phenotypes of colorectal cancer by activating NF-κB signaling pathway [24]. To detect whether DHX9 could regulate the activity of NF-κB signaling pathway in GC, Western Blot analysis showed that DHX9 overexpression upregulated the expression of p-IκB and p-p65. However, co-transfection of DHX9 overexpression and si-ILF3 indicated that DHX9 increased the p-IκB and p-p65 would be reversed by ILF3 knockdown (Fig. 7F). It means the interaction between ILF3 plays a crucial role for DHX9 to activate the NF-κB signaling pathway. Finally, we examined the role of DHRS4-AS1 in formation of the complex between DHX9 and ILF3. CO-IP results showed that DHRS4-AS1 overexpression decreased the ILF3 in co-immunoprecipitation for DHX9 in HGC-27. On the contrary, Knockdown of DHRS4-AS1 led to increased binding of DHX9 to ILF3 in AGS cells (Fig. 7G). Taken together, these results demonstrated that the lncRNA DHRS4-AS1 inhibited interaction between DHX9 and ILF3, thereby inhibiting the activation of NF-κB signaling pathway by DHX9.

## Discussion

GC prognosis is very poor owing to the difficulty of early diagnosis and rapid proliferation [27]. Exploring the molecular biomarkers for early diagnosis of gastric cancer is crucial for improving the therapeutic efficacy of gastric cancer treatment. LncRNAs are novel genes involved in GC progression that had verified in previous study []. Since lncRNAs do not encode proteins by themselves, they usually function by regulating gene transcription, protein translation, or post-translational protein modification [29]. However, whether lncRNAs drive proliferation or apoptosis in GC has not been extensively studied. In this study, we report that lncRNA DHRS4-AS1 plays a vital role in GC proliferation and promoting GC cell apoptosis by inhibiting DHX9. DHRS4-AS1 expression is generally downregulated in GC tissues. Moreover, decreased DHRS4-AS1 expression is significantly associated with poor prognosis in gastric cancer patients.

LncRNA DHRS4-AS1 is a natural antisense transcript of the *DHRS4* gene, which functions as an oncogene in some malignant tumors [30, 31]. Interestingly, lncRNA DHRS4-AS1 exhibit tumor suppression properties. DHRS4-AS1 suppresses cell proliferation and promotes apoptosis via the miR-522-3p/SOCS5 axis In hepatocellular carcinoma [32]. In addition, DHRS4-AS1 inhibits the NSCLC cell stemness and correlates with tumor immune cell infiltration [33]. We show that lncRNA DHRS4-AS1 is highly expressed in normal gastric mucosa tissue and is downregulated in GC tissue. Knocking down or overexpressing DHRS4-AS1 significantly suppresses or enhances cancer cell apoptosis, respectively, and is closely related to proliferation in vitro and in vivo. Overall, our study and previous studies suggest that DHRS4-SA1 functions as a tumor suppressor in many cancer types.

Many studies reported that lncRNAs regulate gene expression. In general, lncRNAs in the cytoplasm act as endogenous RNAs that compete for microRNAs and regulate protein expression [34–36]. Other lncRNAs in the nucleus usually act as scaffolds by interacting with RNA binding protein and regulating chromatin structure [37–39]. In this study, we showed that lncRNA DHRS4-AS1 acts as a scaffold to enhance interactions between the E3 ligase MDM2 and DHX9. MDM2 serves as E3 Ligase regulates cancer cell proliferation in various tumors [40]. DHRS4-AS1 directly binds to DHX9 and negatively regulates DHX9 expression. We also showed that DHRS4-AS1 recruit E3 ligase MDM2 and enhance its associate with DHX9, thereby mediating DHX9 ubiquitination. However, further investigation is required to examine details regarding DHRS4-AS1-mediated DHX9 ubiquitination.

DHX9 functions as a oncoprotein that is closely associated with tumor cell proliferation and metastasis [41, 42]. In this study, we also observed that DHX9 is significantly upregulated in GC tissues. DHX9 expression in GC tissues is associated with patient prognosis. Furthermore, DHX9 promotes GC cell proliferation and inhibits apoptosis. Moreover, DHX9-mediated effects on GC cell proliferation and apoptosis are reversed by lncRNA DHRS4-AS1. Those results suggested that lncRNA DHRS4-AS1 that the effect of DHRS4-AS1 on gastric cancer proliferation and apoptosis is achieved through targeted downregulation of DHX9. To further explore the molecular mechanism of DHX9 on the malignant behavior of gastric cancer, we found that DHX9 interacts with a arcinogenesis ILF3 which was also reported contributed to the malignant phenotypes of multiple tumors. Moreover, Further map the regions of DHX9 and ILF3 demonstrated that the signature protein domains Myc 3 residues 636–1200 of DHX9 responsible for interaction with domains Flag 1 residues 1-378 and Flag 3 residues 592–894 of ILF3. This provides a potential target for our future therapy targeting DHX9. In addition, previous studies demonstrated that DHX9 was required for NF-κB mediated transcriptional activation [43–45]. NF-κB activation is constantly observed in GC and correlated with cellular processes including proliferation, apoptosis and metastasis [46]. Accumulating evidence indicates that DHX9 functions as a driver of carcinogenesis and showed positively correlated with NF-κB signal activation. We also found that the expression level of ILF3 was significant up-regulated in DHX9 overexpressed GC cells. Moreover, we found the associate between DHX9 and ILF3 was crucial for activation of NF-κB signal pathway in GC. Knockdown the expression of ILF3 in DHX9 overexpressed GC cell significant suppressed the activation of NF-κB. It indicated that the activation of NF-κB by DHX9 in GC was an ILF3-depend manner. Furthermore, our study verified that lncRNA DHRS4-AS1 overexpressed significant inhibited the associate between of ILF3 and DHX9 while DHRS4-AS1 enhanced interaction between these two protein. thereby inhibiting the activation of NF-κB signaling pathway by DHX9. Therefore, our work further interprets the molecular mechanism of LncRNA DHRS4-AS1 in gastric cancer, providing new possibilities for clinical application.

In summary, our results illustrate that lncRNA DHRS4-AS1 is a tumor suppressor in GC. DHRS4-AS1 expression is significantly associated with GC prognosis. Moreover, lncRNA DHRS4-AS1 regulates GC apoptosis and cell proliferation by destabilizing DHX9. At the same time, lncRNA DHRS4-AS1 acts as a suppressor to inhibit interactions between the DHX9 and ILF3, thereby interfering with the activation of the NF-κB signaling pathway. Our study reveals new insights into the mechanisms driving GC cell proliferation and apoptosis and highlights the potential of lncRNAs as future therapeutic targets.

## Conclusions

DHRS4-AS1 was significant down-regulated in gastric cancer tissue and low expression of DHRS4-AS1 was correlated with malignant phenotypes and worse outcomes in GC. DHRS4-AS1 acts as a scaffold to facilitate interactions between DHX9 and the E3 ligase MDM2, thus accelerating DHX9 degradation via ubiquitin-proteasome system. DHRS4-AS1 inhibited association between DHX9 and ILF3 and inhibiting the activation of NF-κB signaling pathway by DHX9.

### Electronic supplementary material

Below is the link to the electronic supplementary material.

Supplementary Material 1

Supplementary Material 2

## Data Availability

The datasets used or analyzed in this study are available from the corresponding author on reasonable request.

## Abbreviations

LncRNA: Long noncoding RNA
GC: Gastric cancer
OS: Overall survival
PFS: Progression-free survival
OD: Optical density
IHC: Immunohistochemistry
ISH: In situ hybridization
CHX: Cycloheximid
PBS: Phosphate buffer saline
TCGA: The cancer Genome Project
