# Supplementary material for: DHRS4-AS1 regulate gastric cancer apoptosis and cell proliferation by destabilizing DHX9 and inhibited the association between DHX9 and ILF3
Source: Cancer Cell Int. 2023 Dec 1;23:304. doi: 10.1186/s12935-023-03151-x (PMC10693172; doi:10.1186/s12935-023-03151-x)

Figure 2

Figure 2E

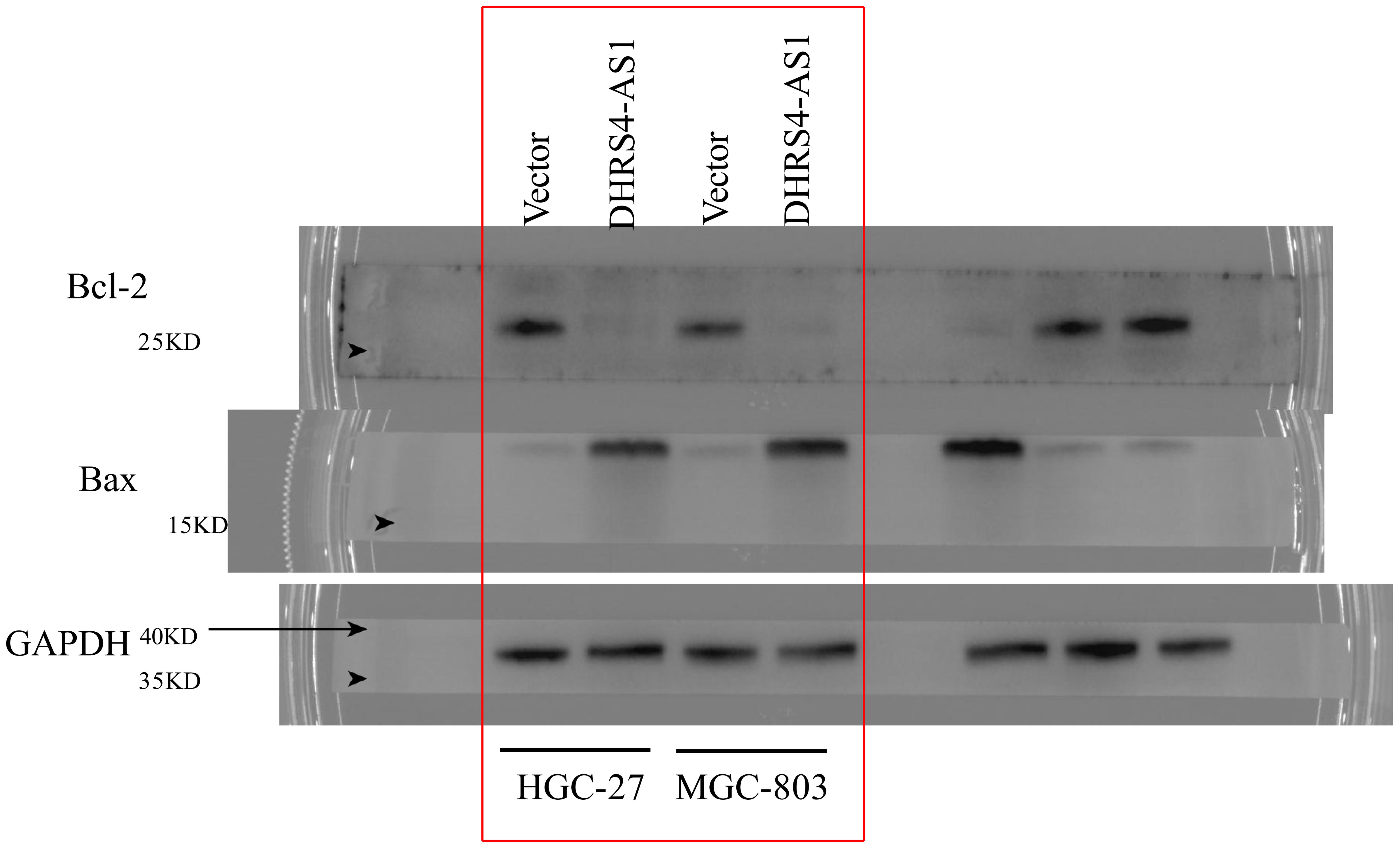

Figure 2J

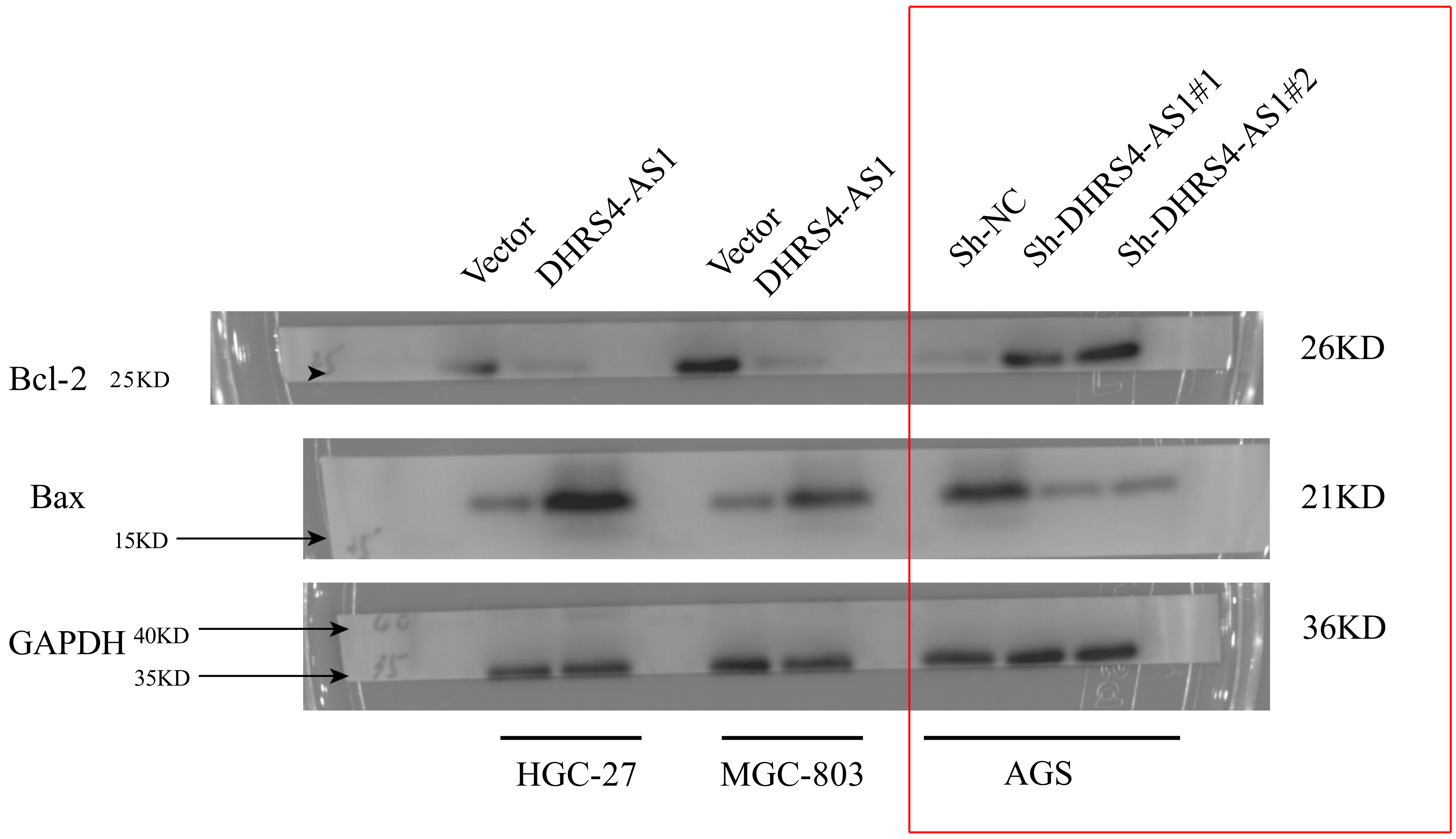

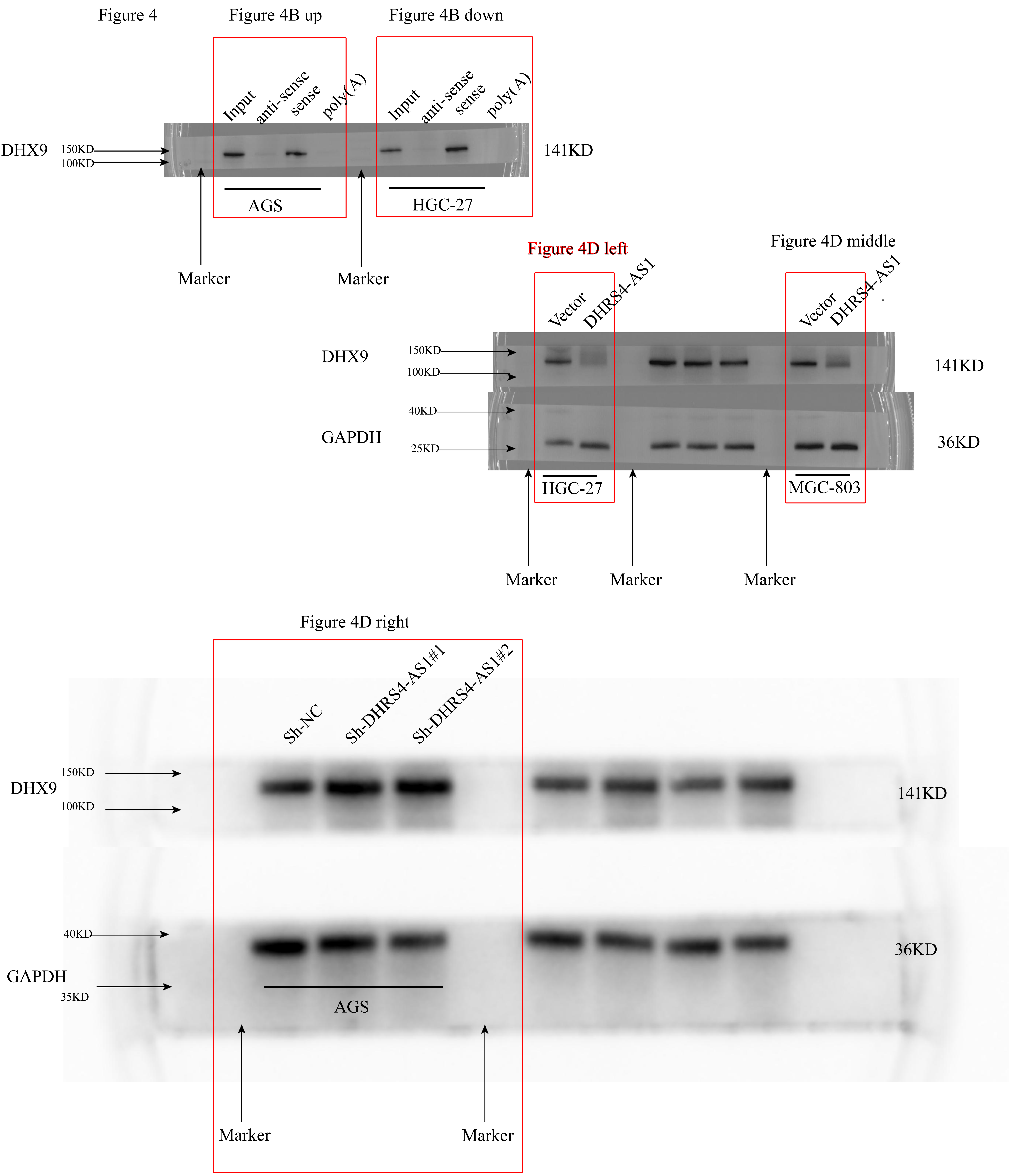

Figure 4
Figure 4B up
Figure 4B down
Input
anti-sense
sense
poly(A)
Input
anti-sense
sense
poly(A)
DHX9
150KD
100KD
141KD
AGS
HGC-27
Marker
Marker
Figure 4D left
Figure 4D middle
Vector
DHRS4-AS1
Vector
DHRS4-AS1
DHX9
150KD
100KD
141KD
40KD
GAPDH
25KD
36KD
HGC-27
MGC-803
Marker
Marker
Marker
Figure 4D right
Sh-NC
Sh-DHRS4-AS1#1
Sh-DHRS4-AS1#2
150KD
DHX9
100KD
141KD
40KD
GAPDH
35KD
36KD
AGS
Marker
Marker

Figure 4G down   Figure 4F Left

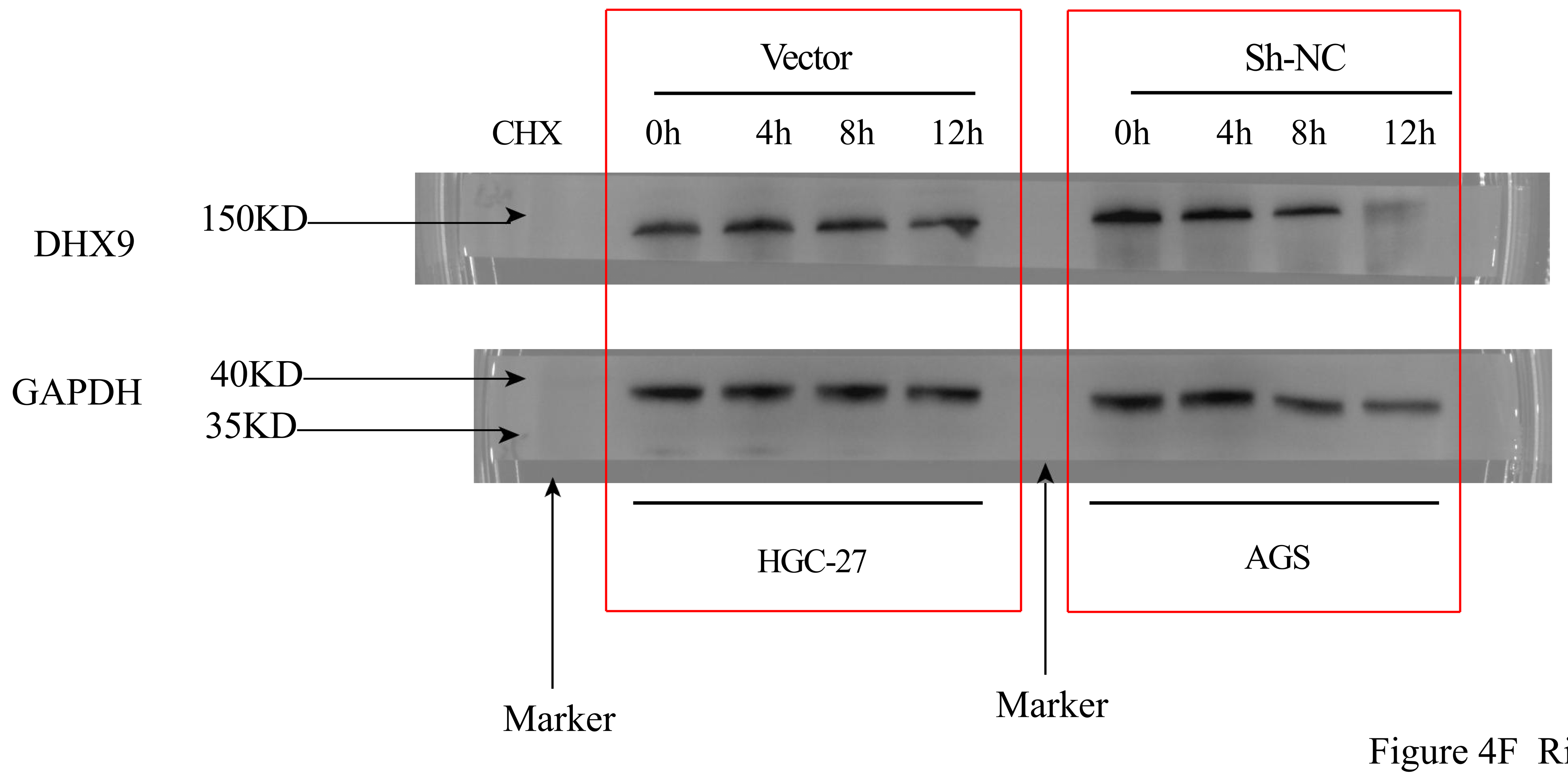

Figure 4F Right

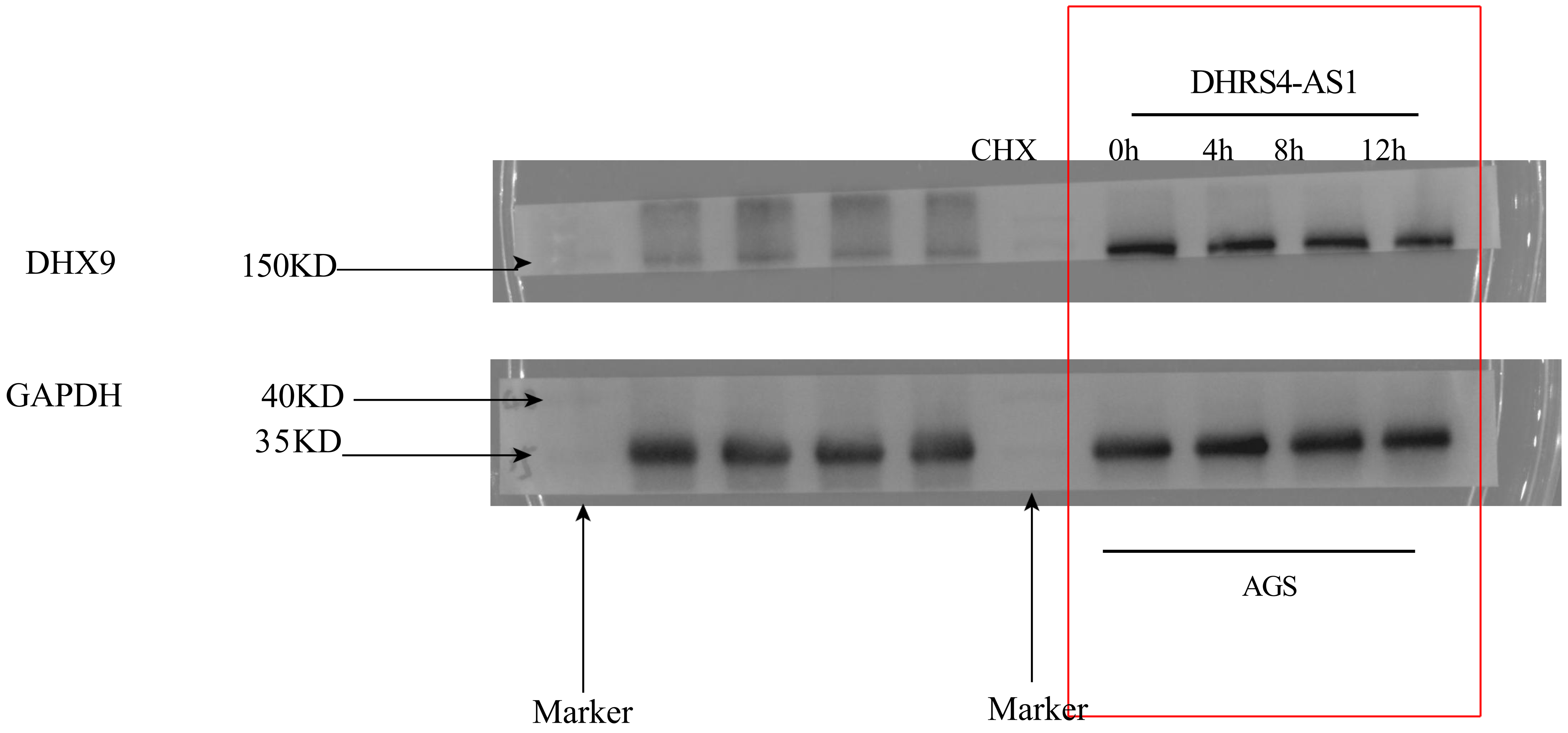

Figure 4G down

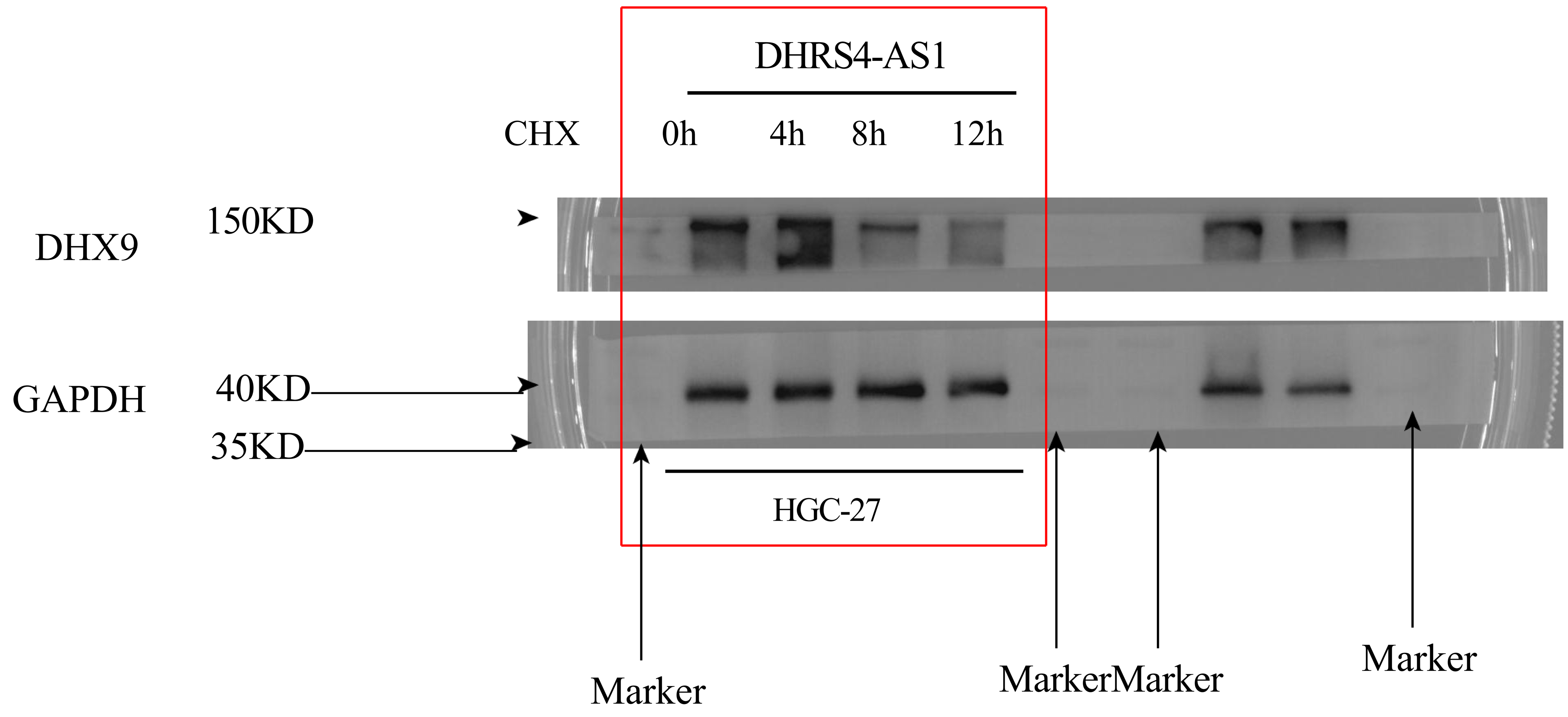

Figure 4G Up

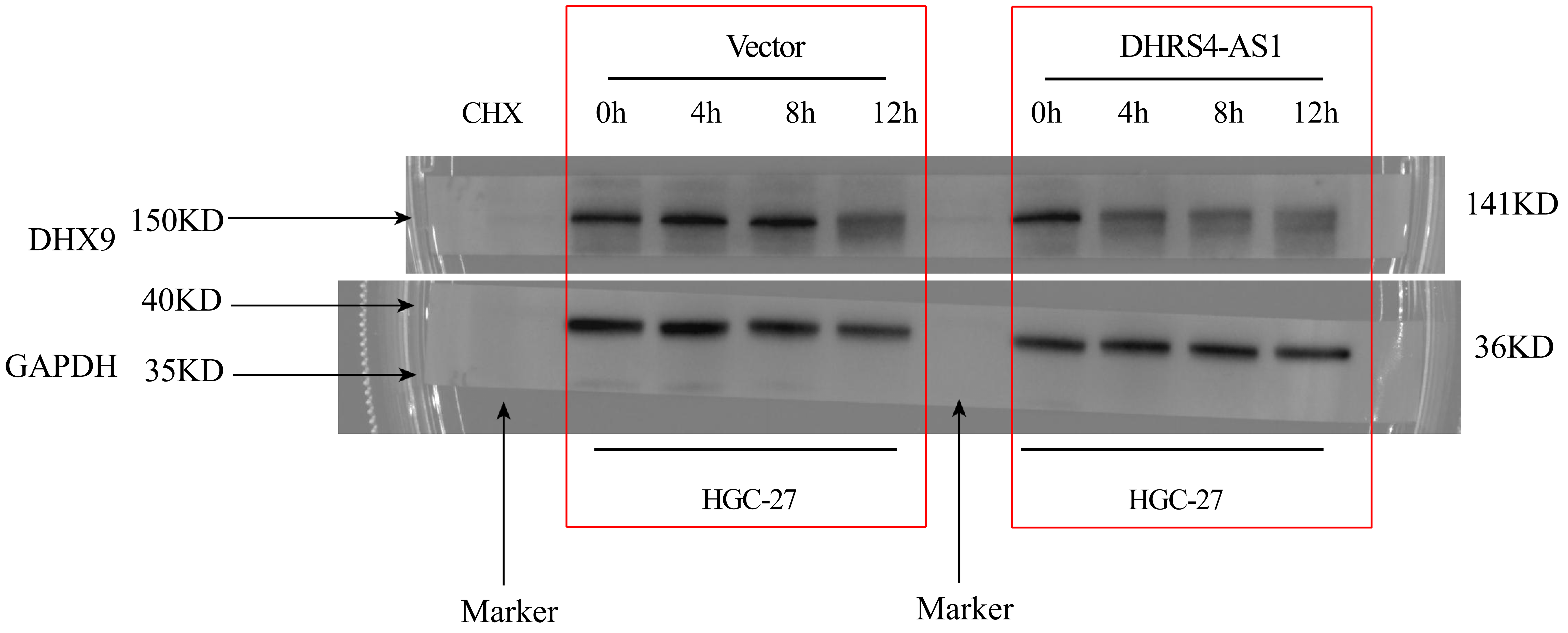

Figure 4H left

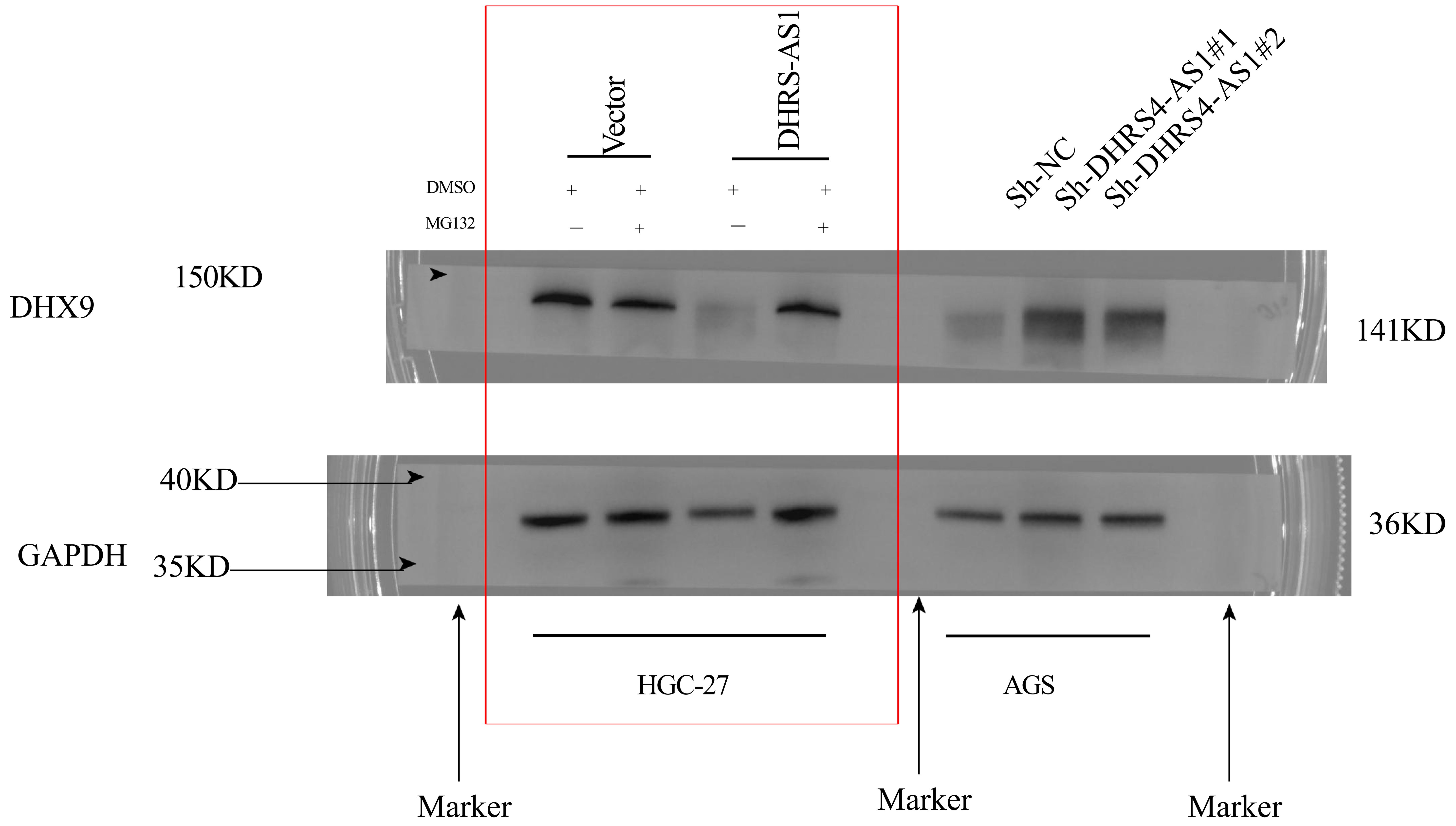

Figure 4H right

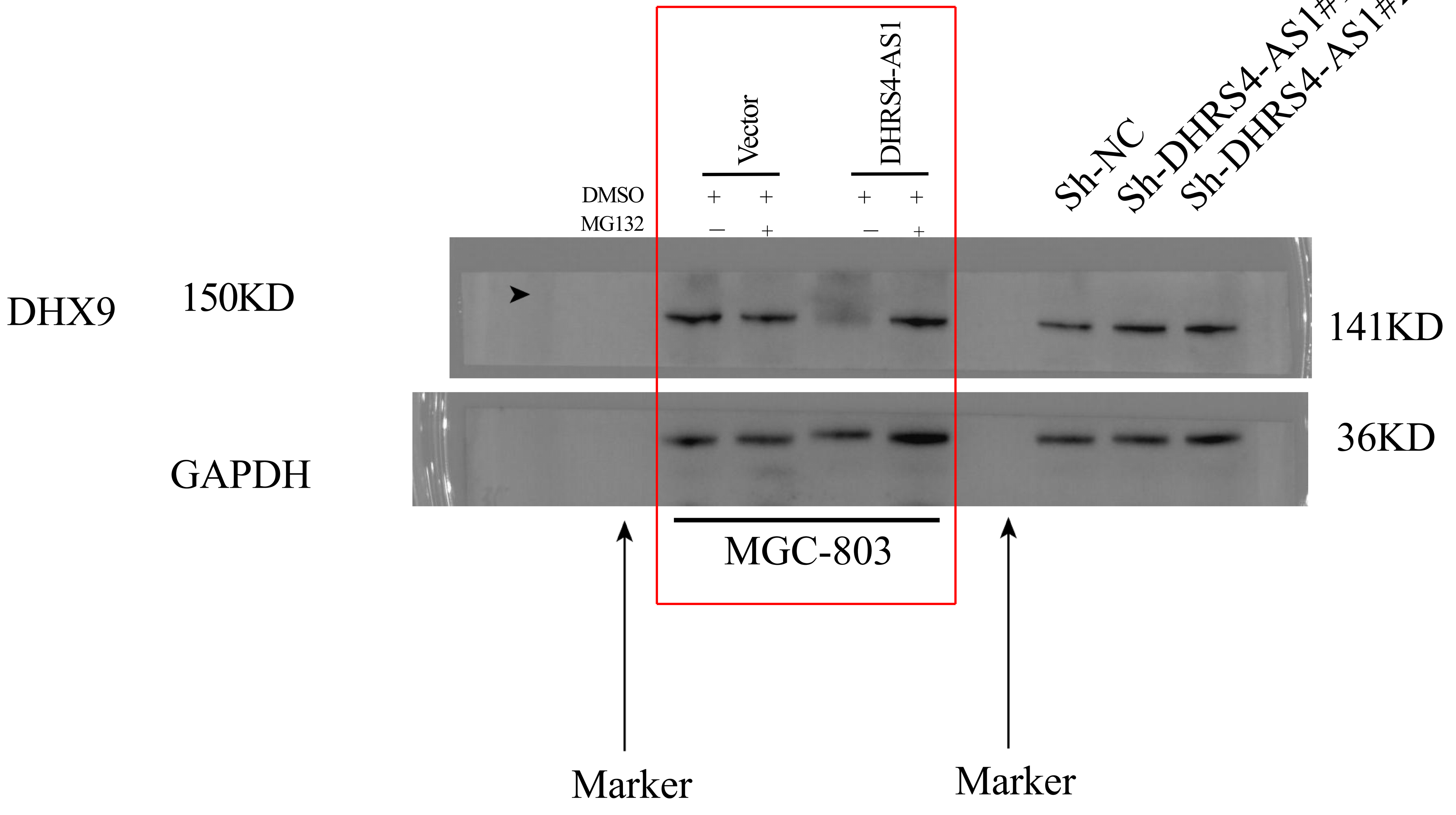

Figure 4I

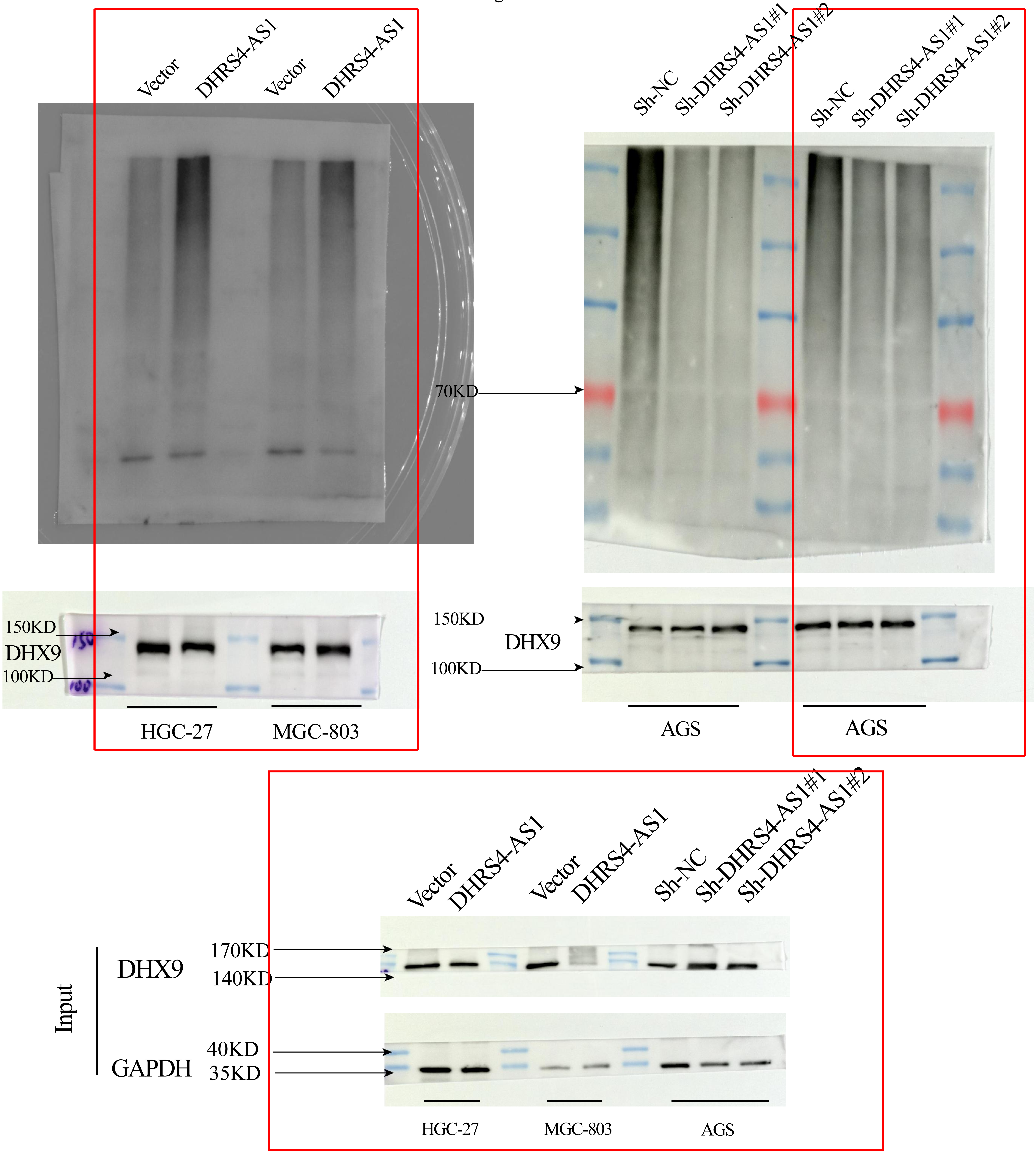
Vector
DHRS4-AS1
Vector
DHRS4-AS1
Sh-NC
Sh-DHRS4-AS1#1
Sh-DHRS4-AS1#2
Sh-NC
Sh-DHRS4-AS1#1
Sh-DHRS4-AS1#2
70KD
150KD
DHX9
100KD
150KD
DHX9
100KD
HGC-27
MGC-803
AGS
AGS
Vector
DHRS4-AS1
Vector
DHRS4-AS1
Sh-NC
Sh-DHRS4-AS1#1
Sh-DHRS4-AS1#2
Input
DHX9
170KD
140KD
GAPDH
40KD
35KD
HGC-27
MGC-803
AGS

Figure 5

Figure 5B

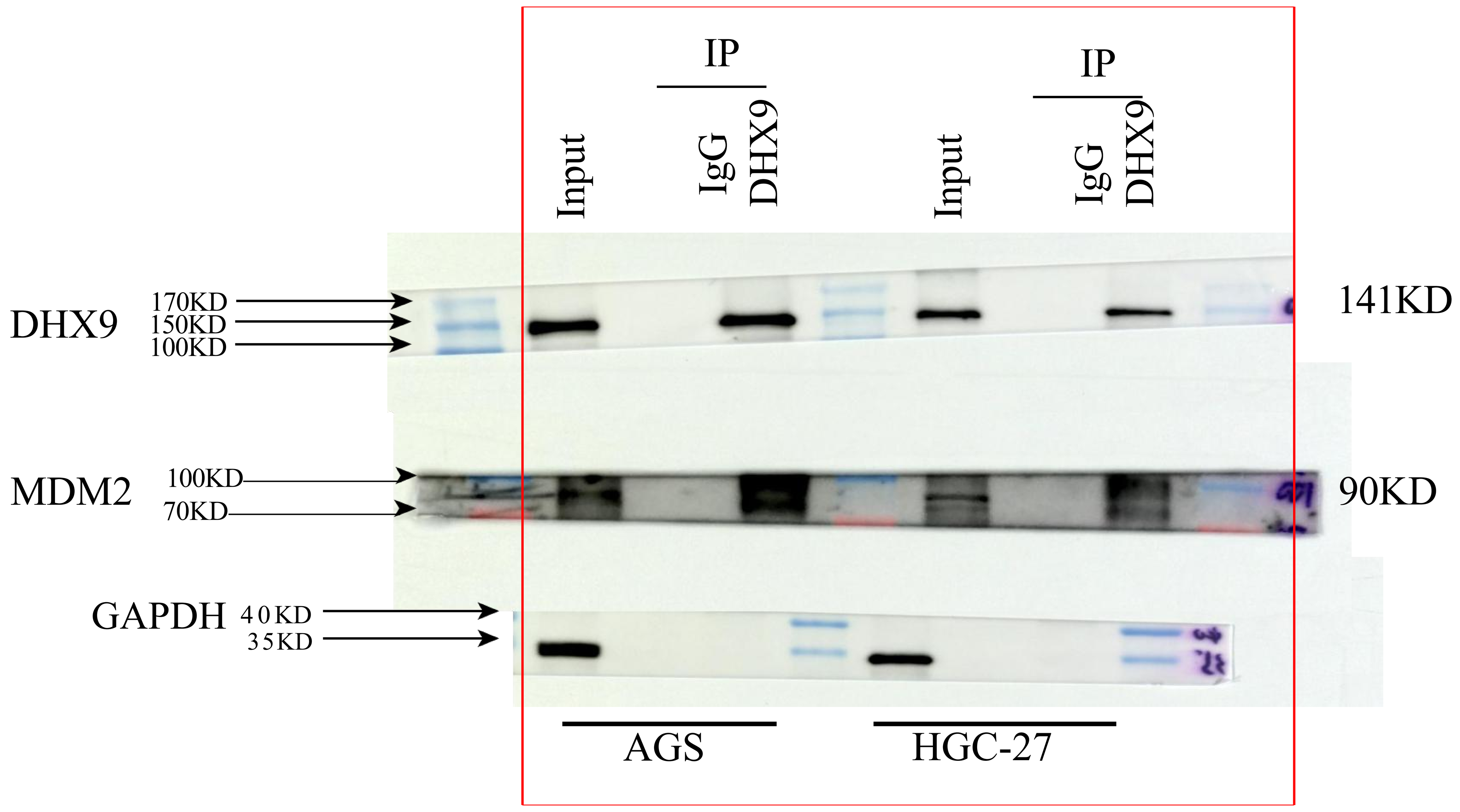

Figure 5C

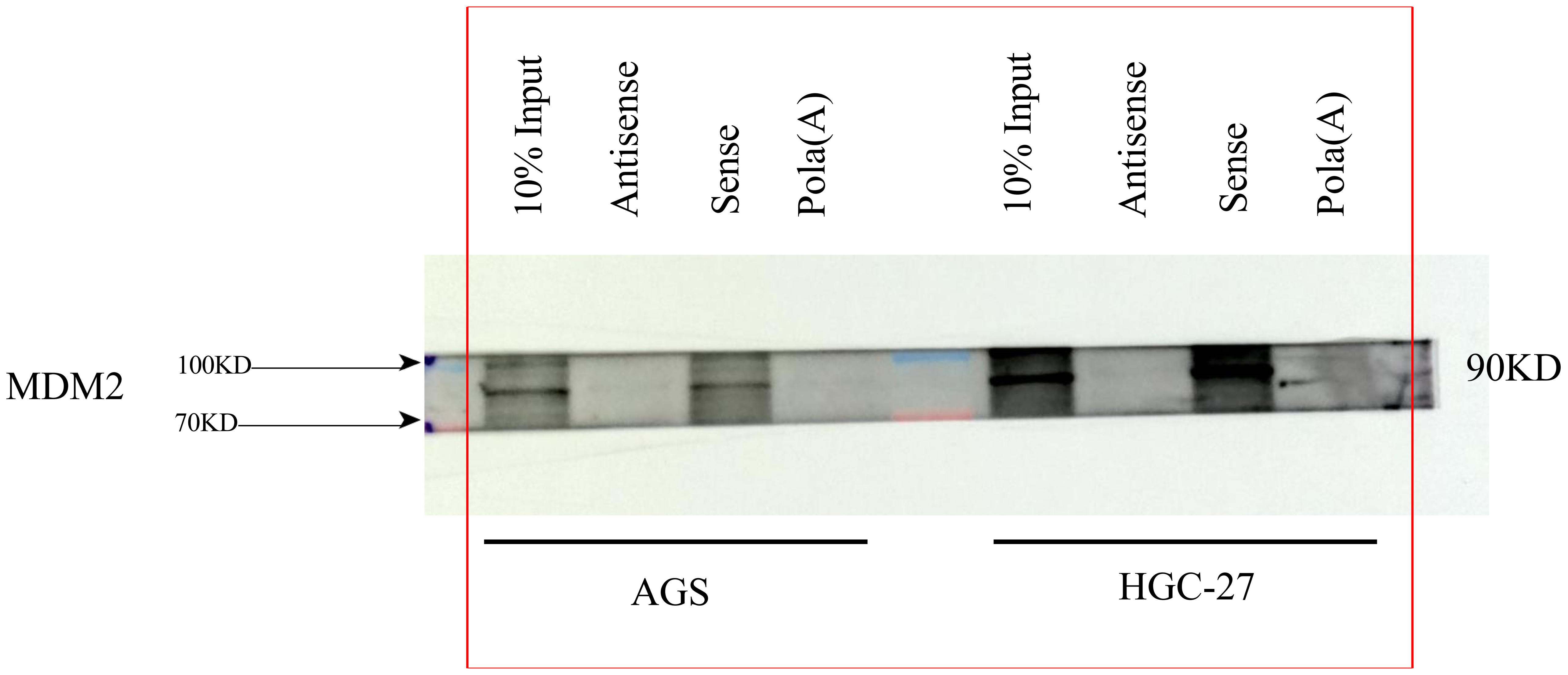

Figure 5E

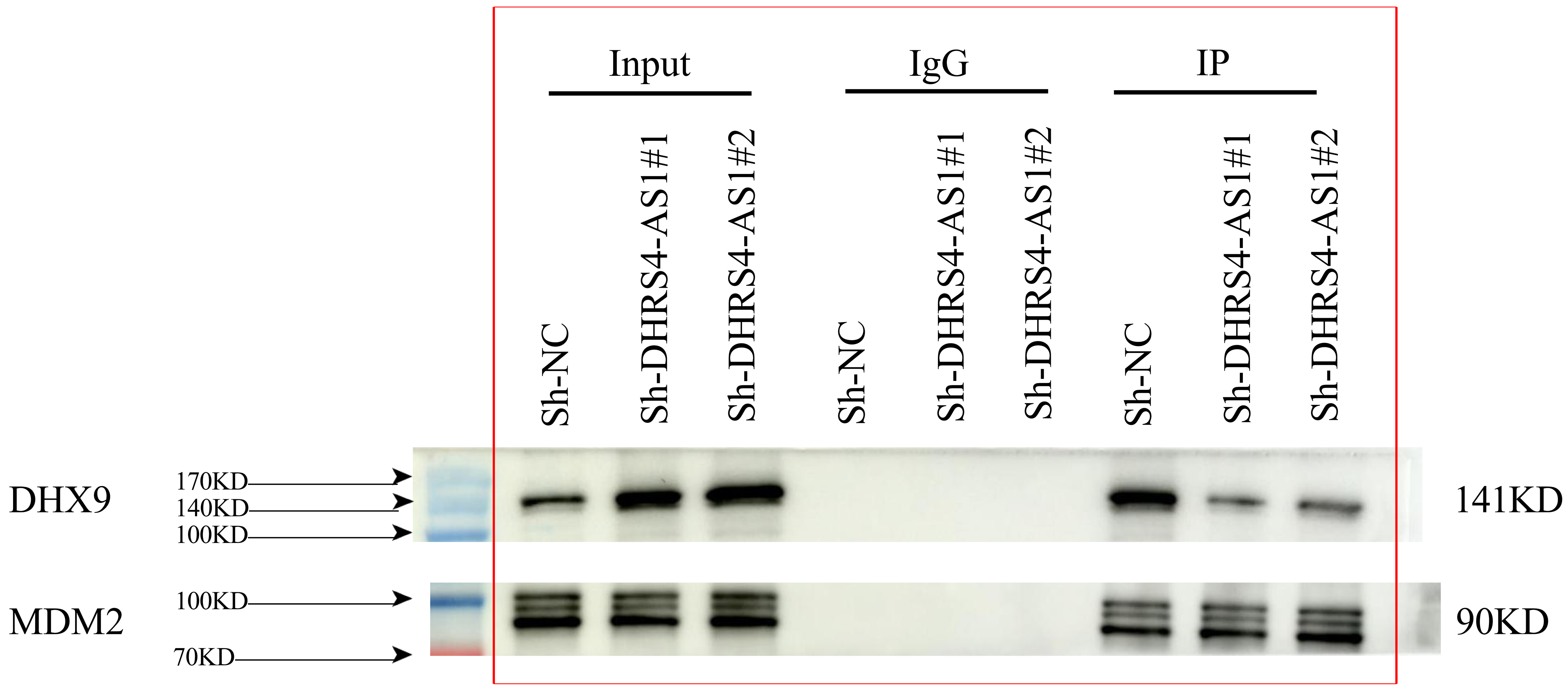

Figure 5F

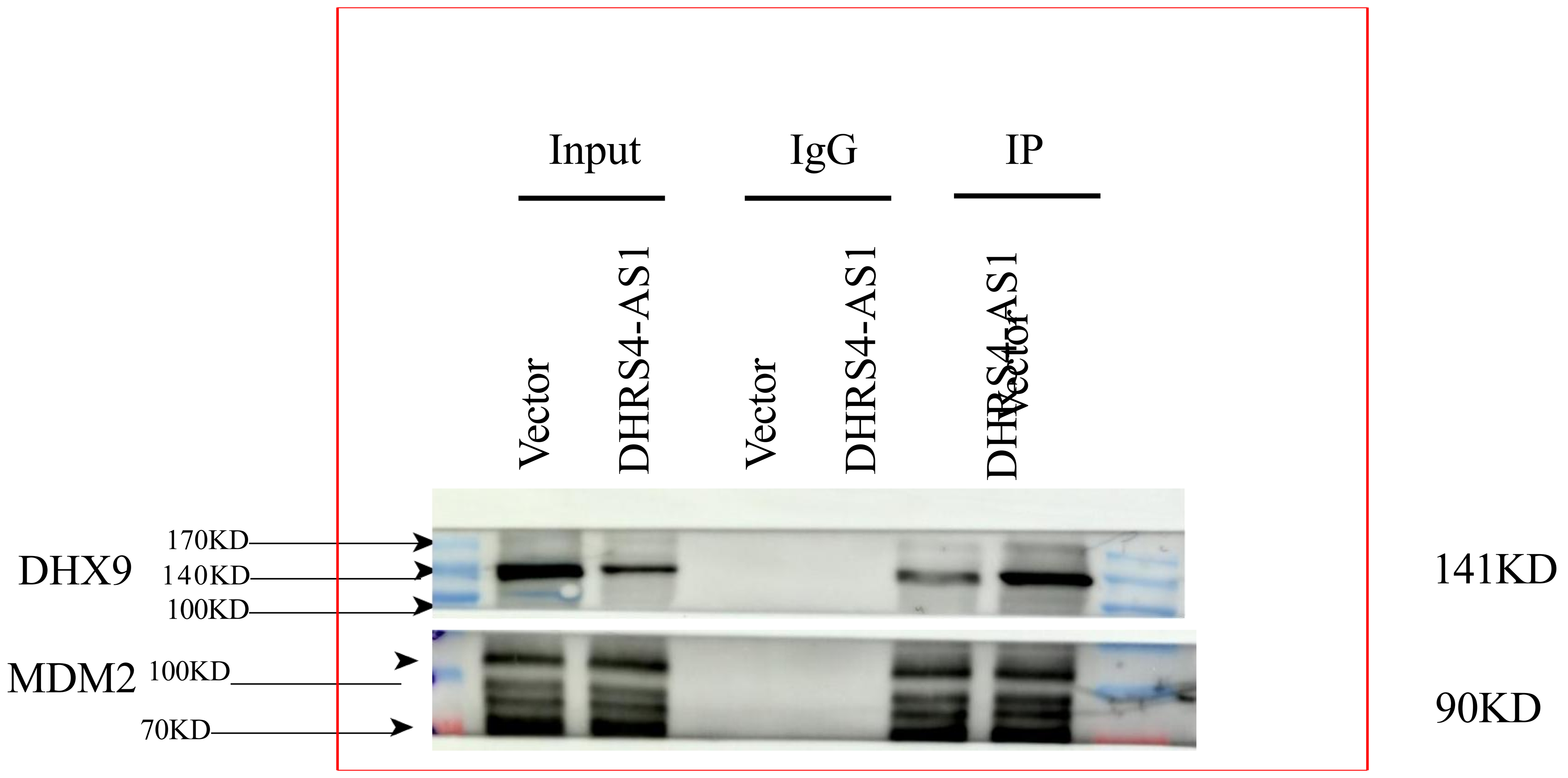

Figure 6

Figure 6C

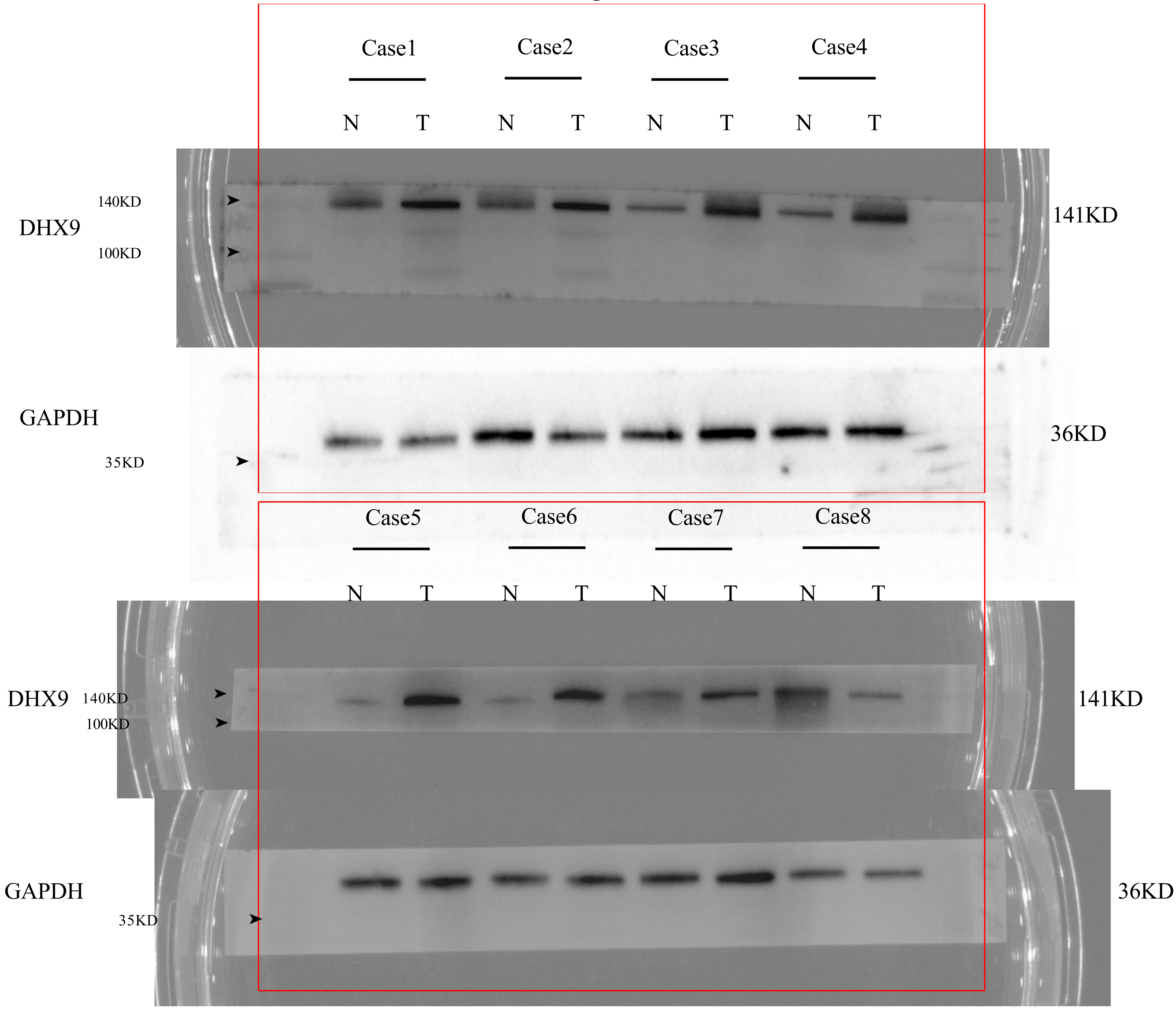

Figure 6

Figure 6H

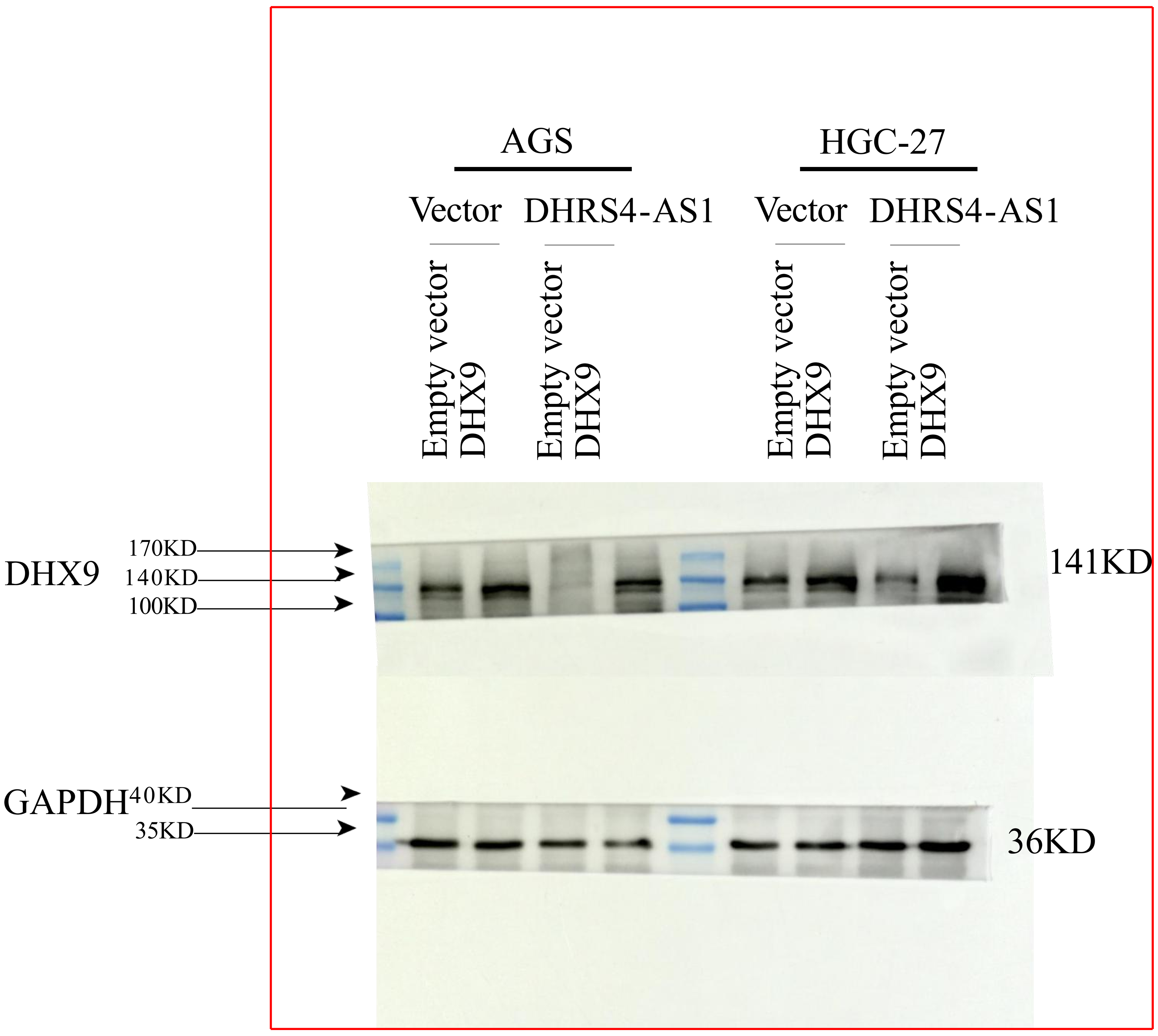

Figure 7

Figure 7A Left

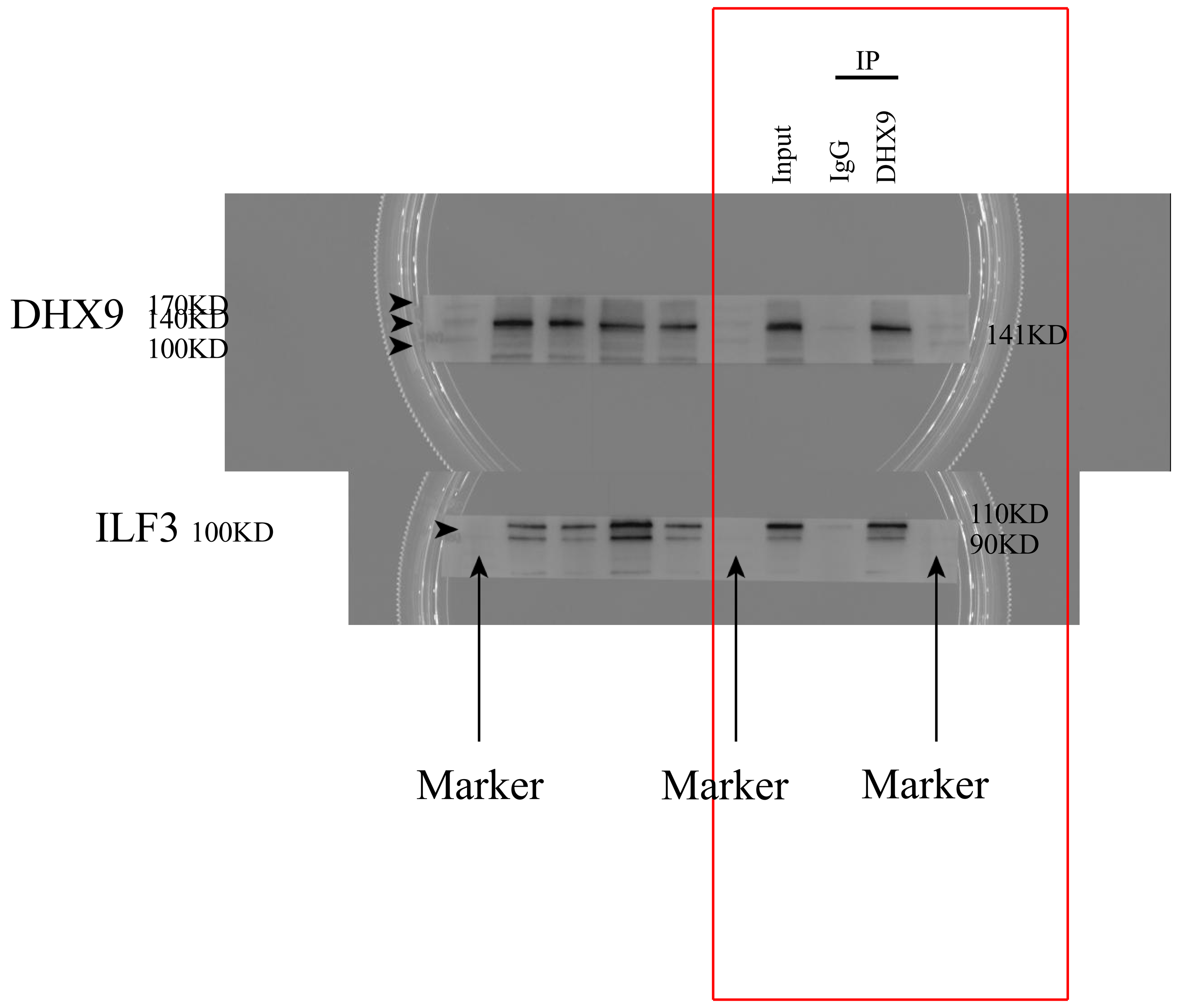

Figure 7

Figure 7A Right

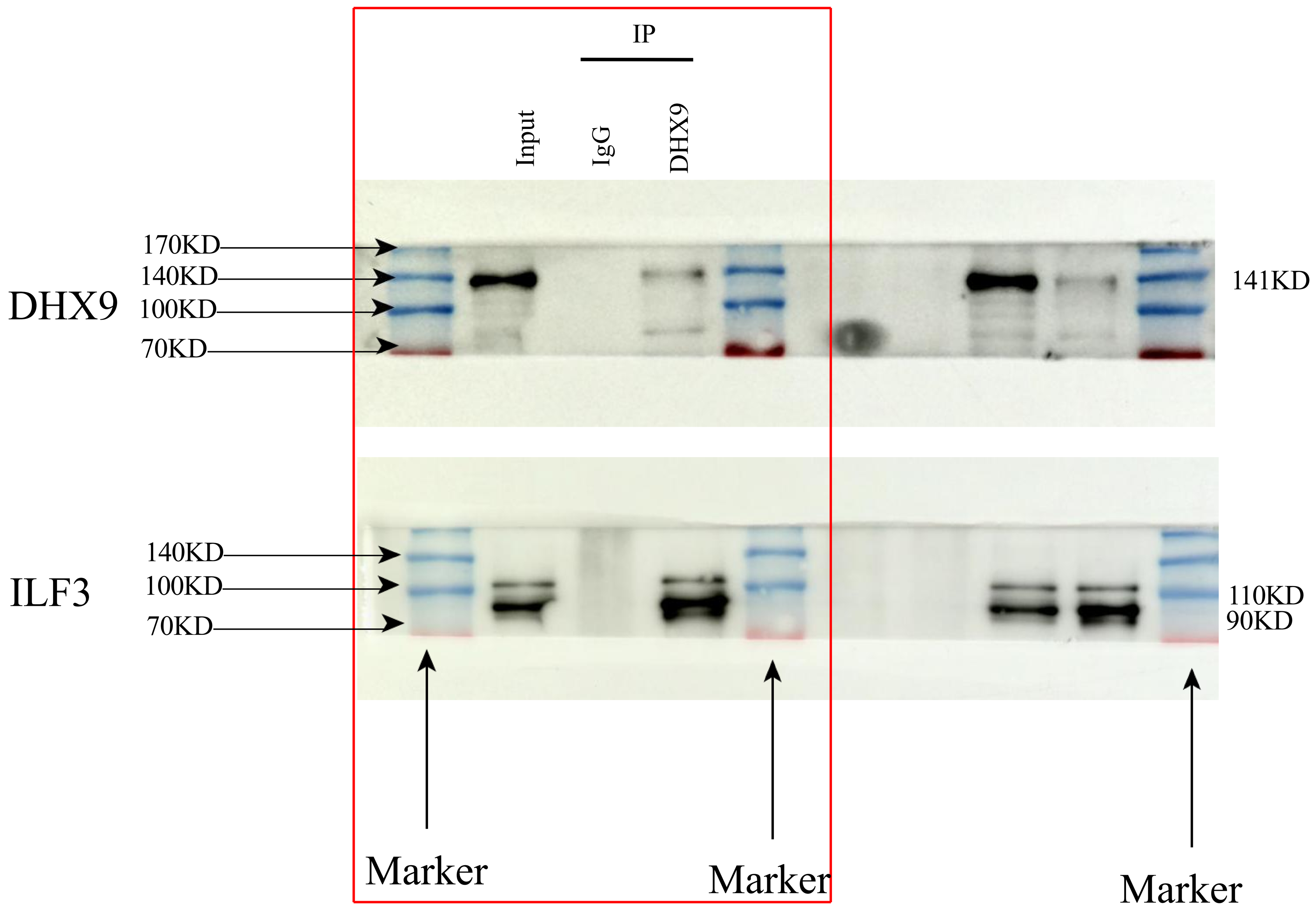

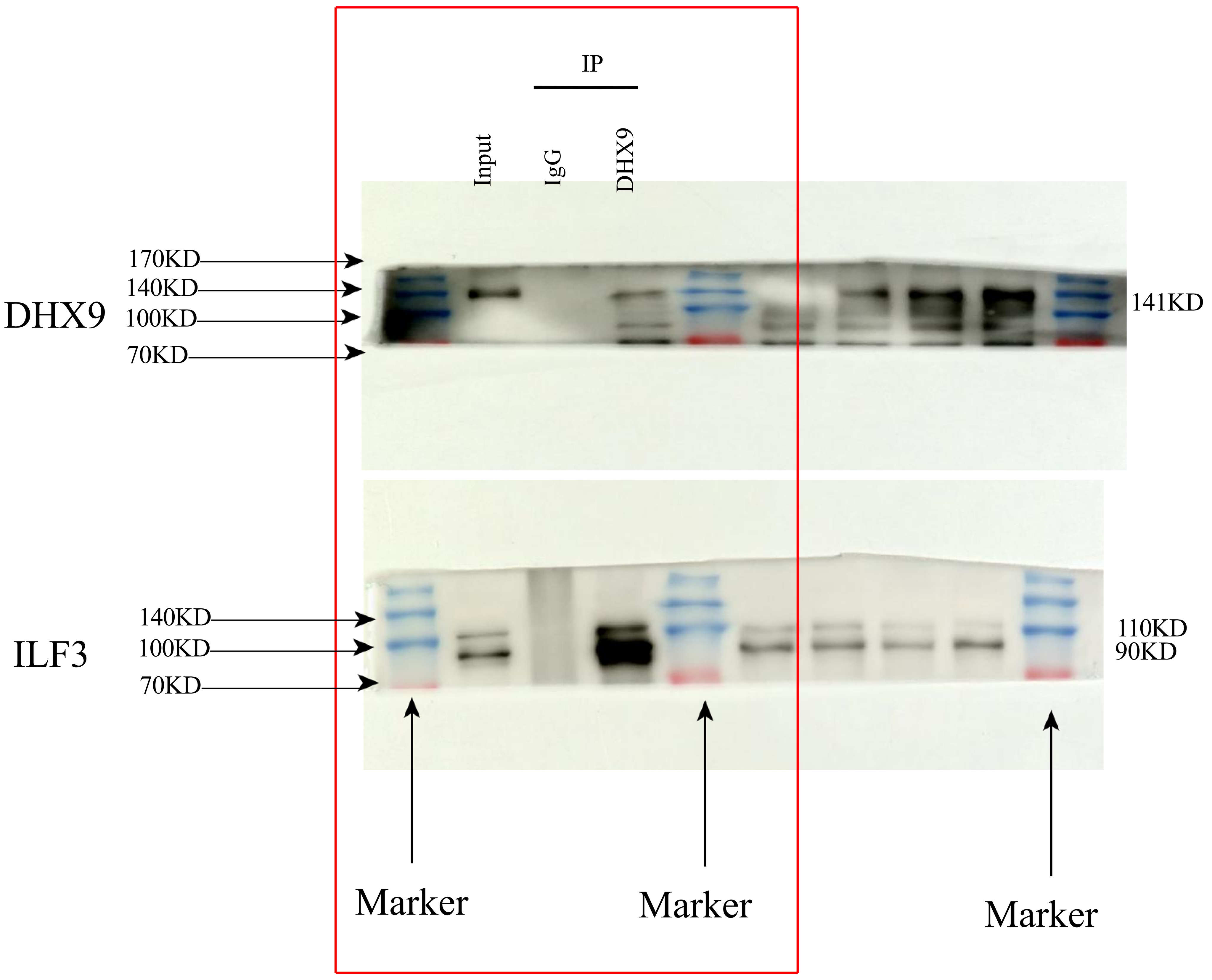

Figure 7

Figure 7B

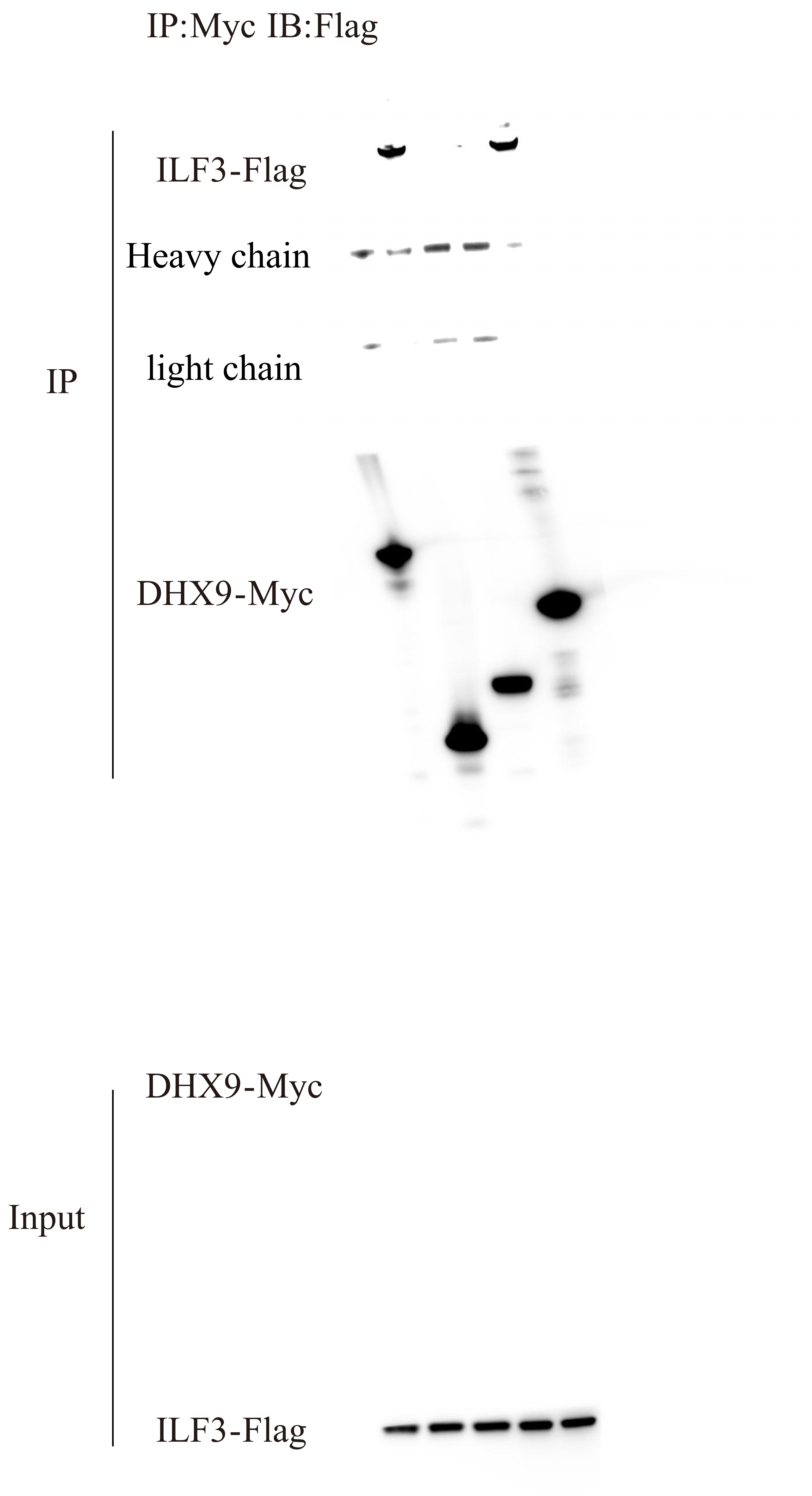

Figure 7

Figure 7C

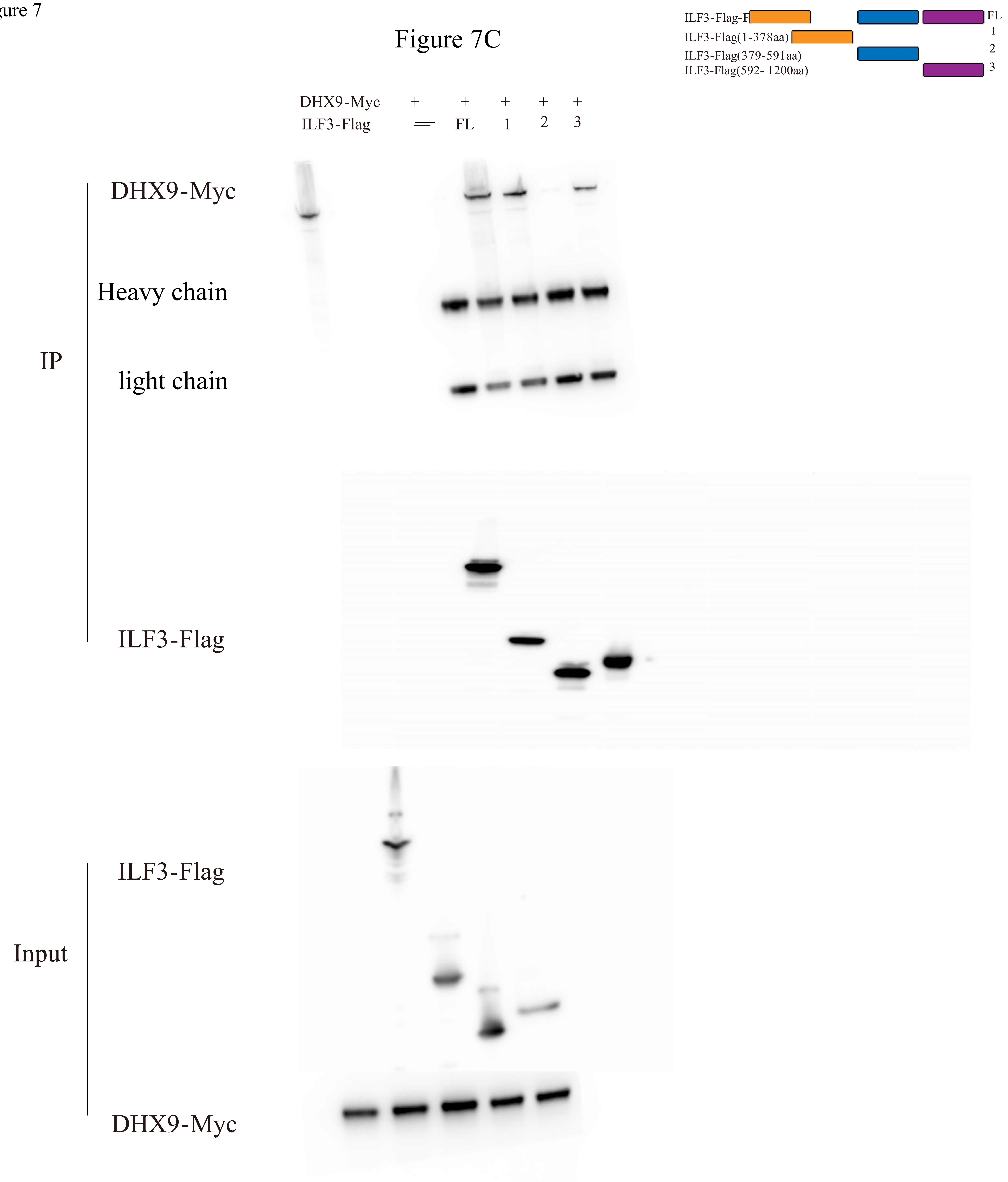

Figure 7

Figure 7F

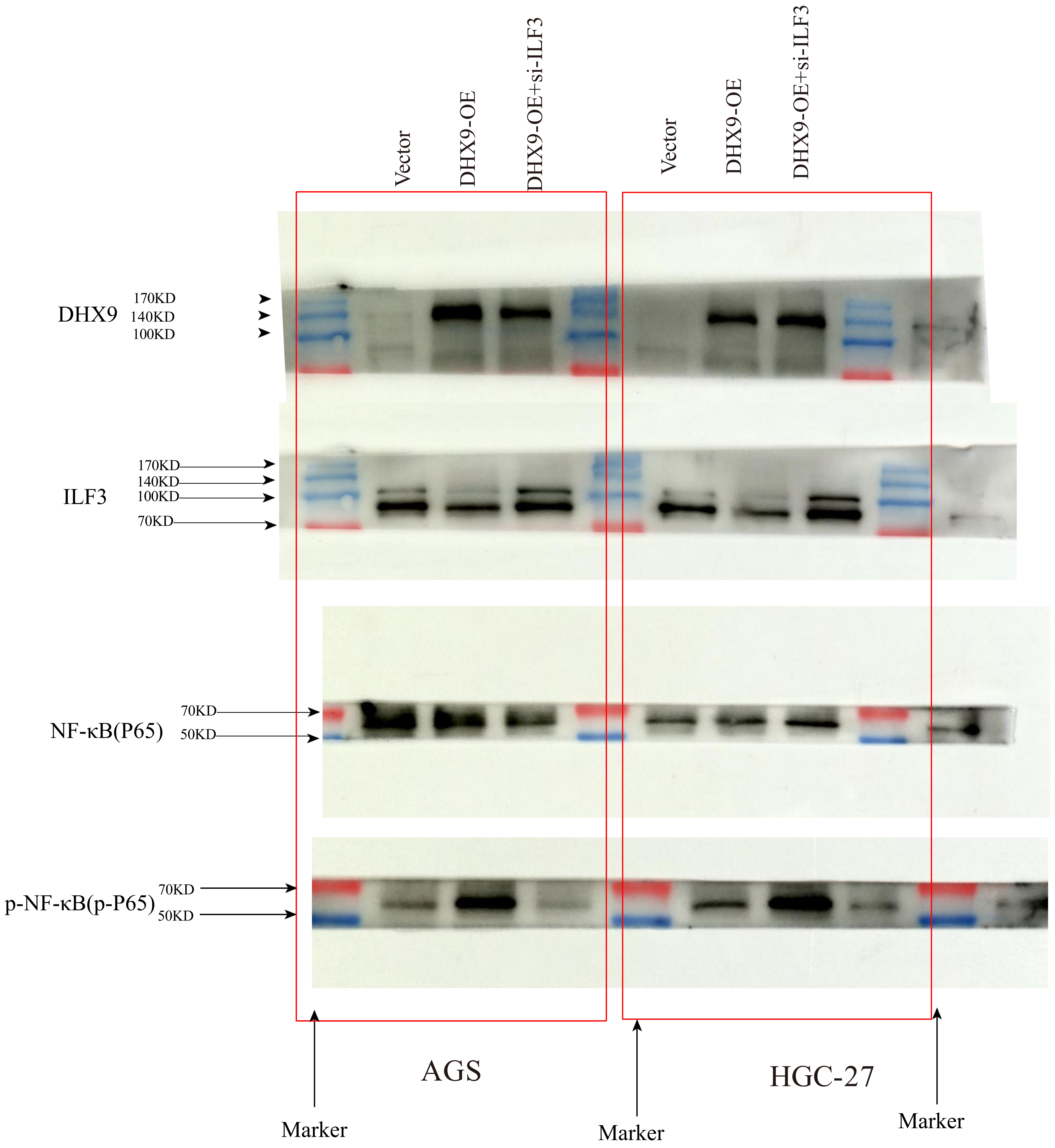

Figure 7

Figure 7F

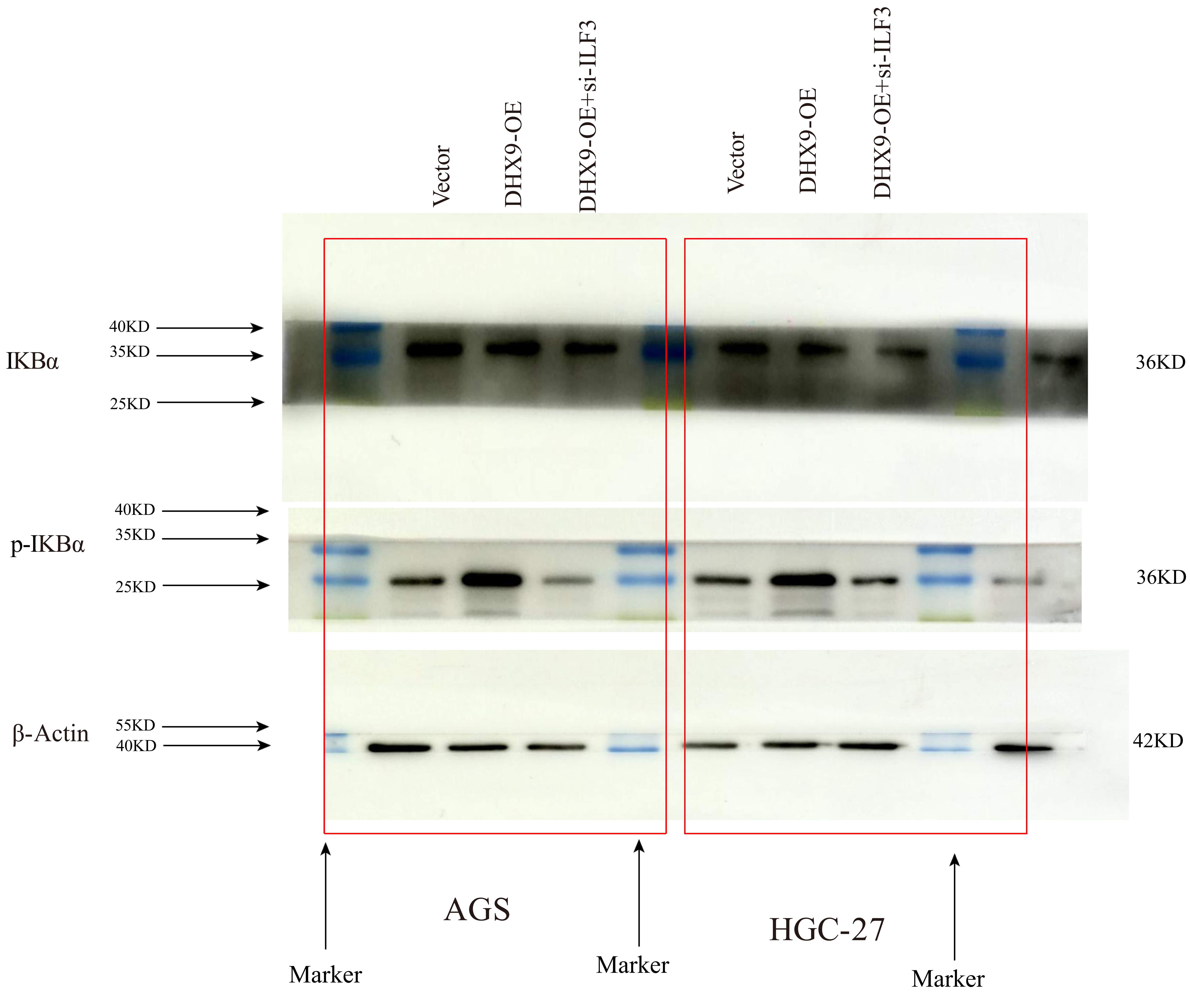

Figure 7

Figure 7G Right

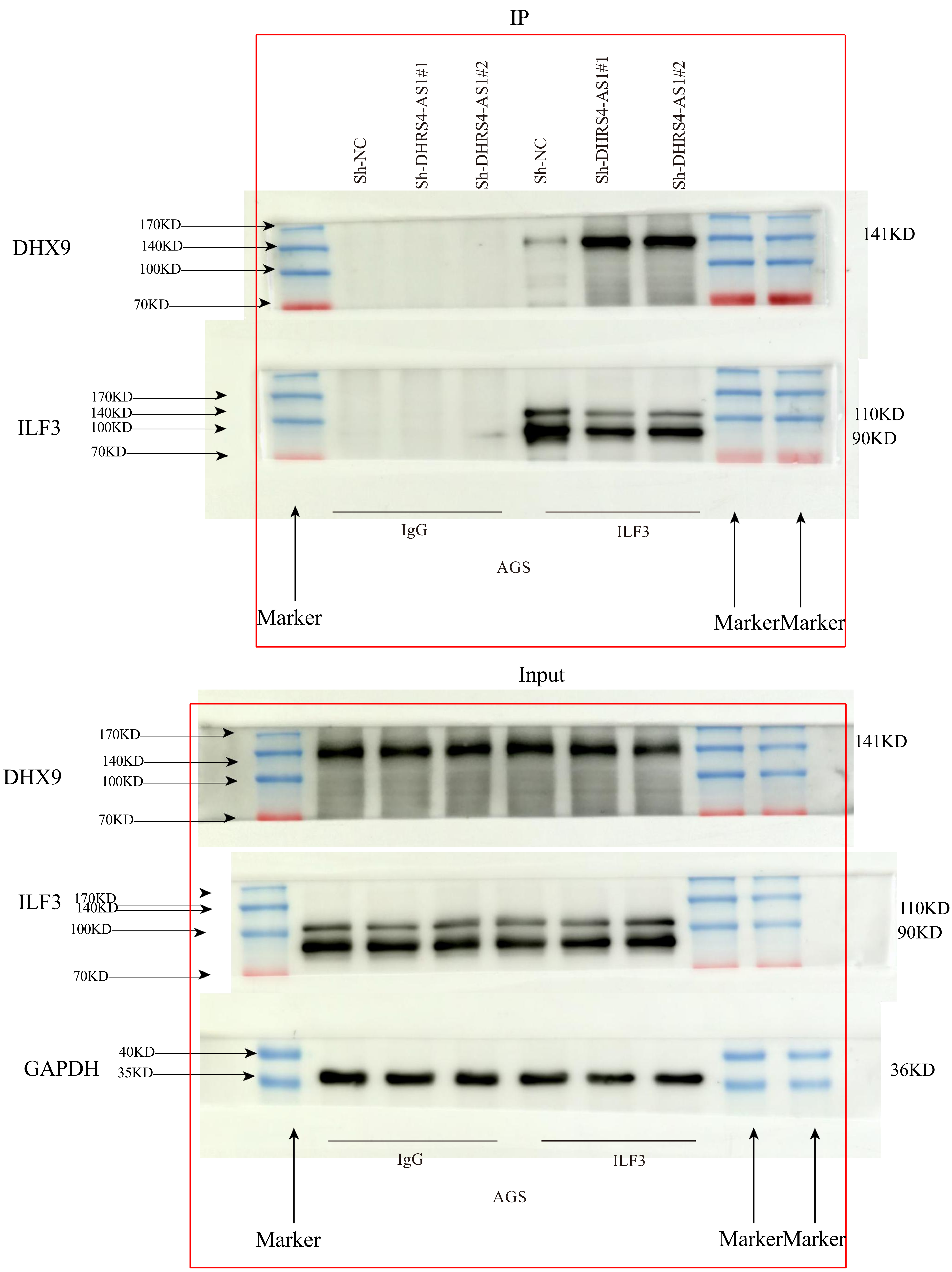

Figure 7

Figure 7G Right

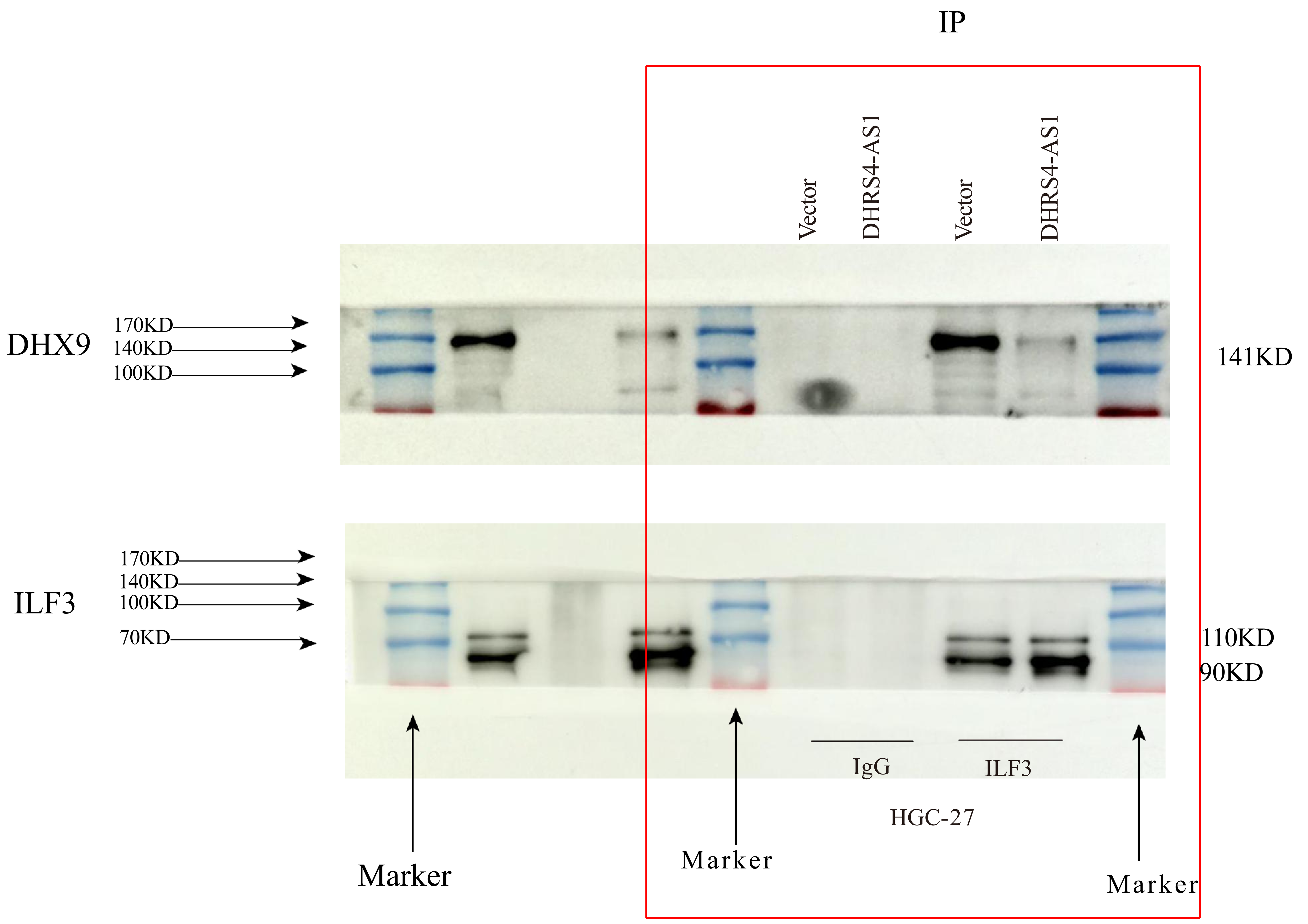

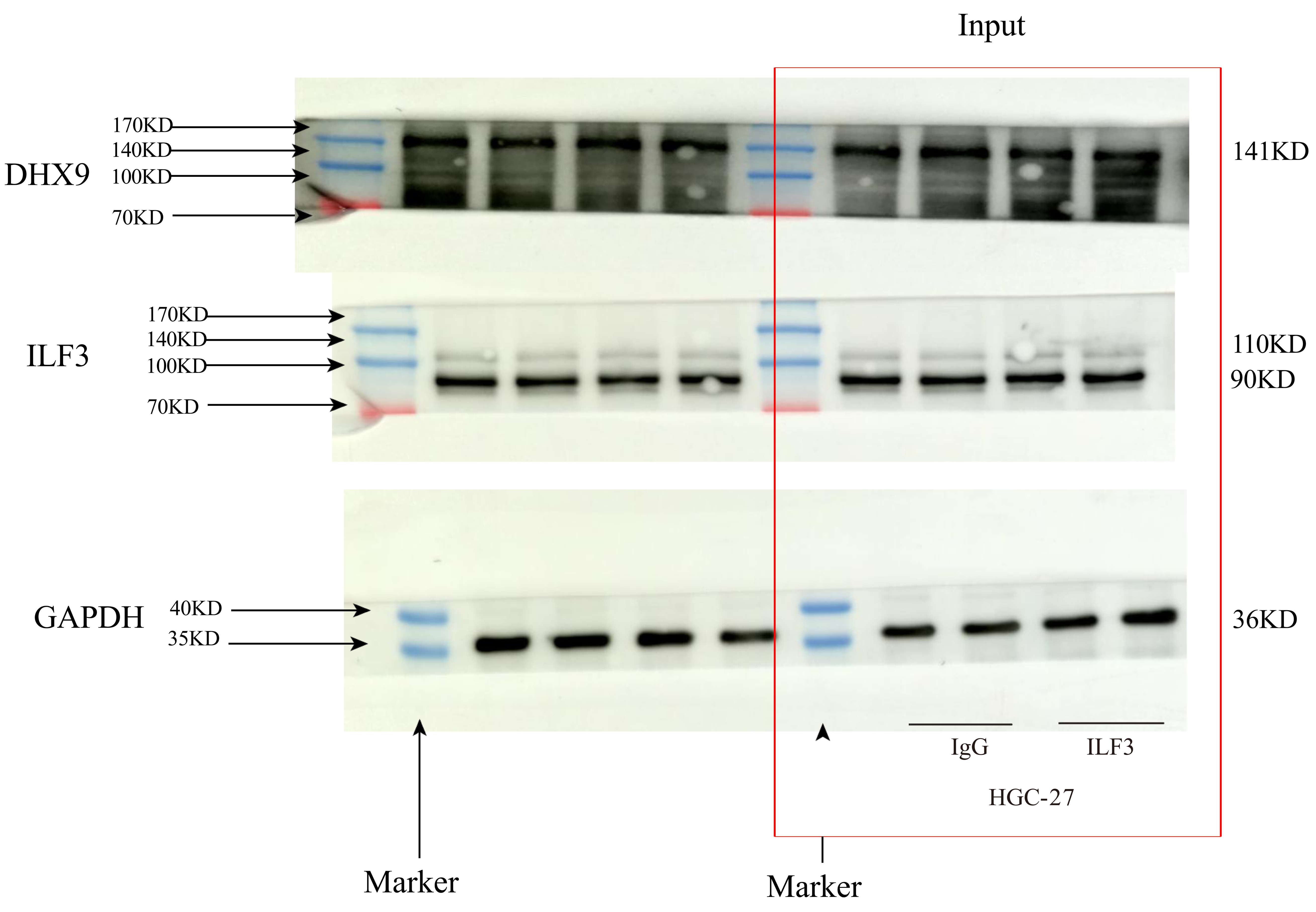

Supplement: Supplementary file 1 — Supplementary Material 1 [file 12935_2023_3151_MOESM1_ESM.pdf]
