# Supplementary material for: DHRS4-AS1 regulate gastric cancer apoptosis and cell proliferation by destabilizing DHX9 and inhibited the association between DHX9 and ILF3
Source: Cancer Cell Int. 2023 Dec 1;23:304. doi: 10.1186/s12935-023-03151-x (PMC10693172; doi:10.1186/s12935-023-03151-x)
Supplement: Supplementary file 2 — Supplementary Material 2 [file 12935_2023_3151_MOESM2_ESM.docx]

**
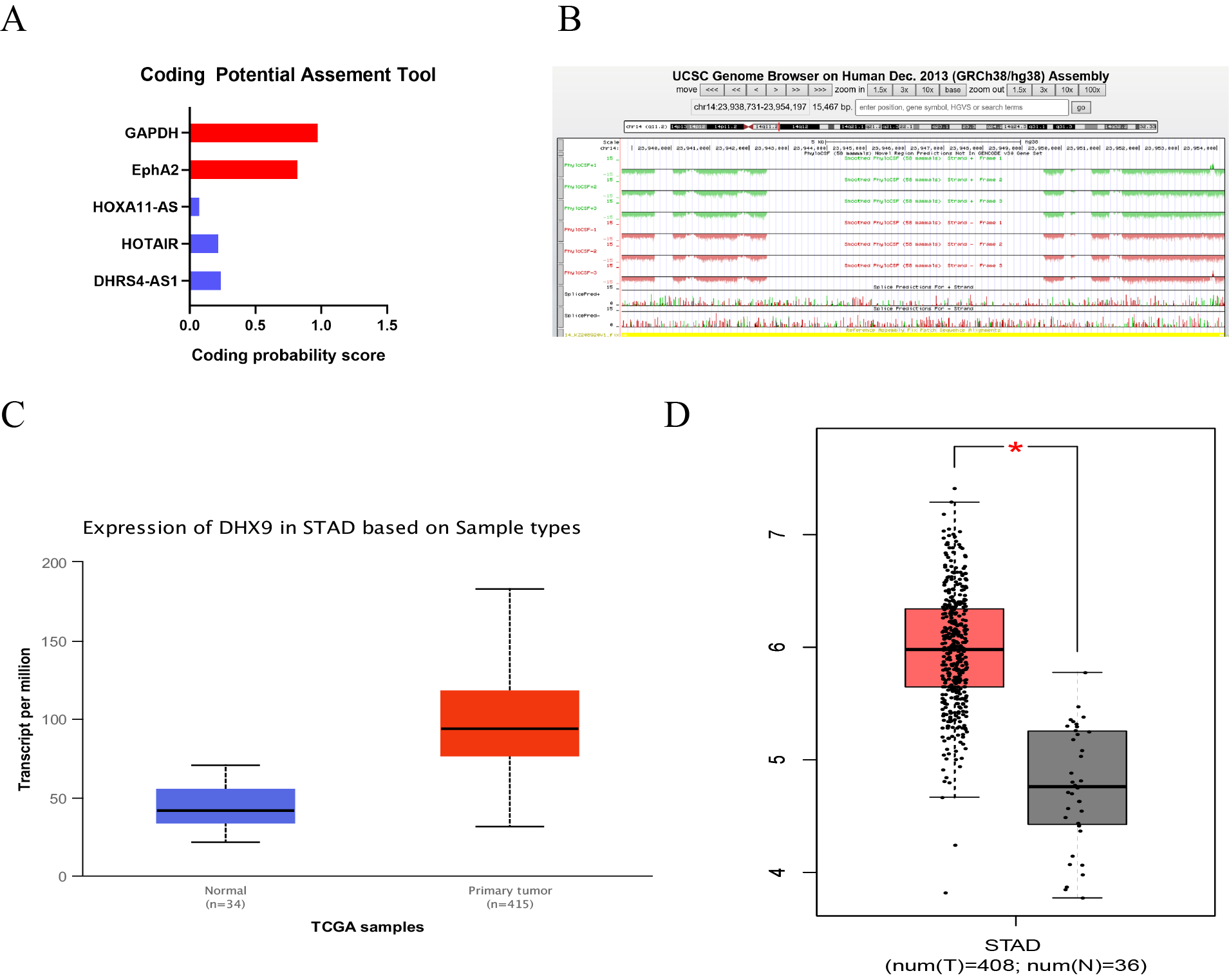
**

Fig. S1: The prediction of protein-coding potential of lncRNA DHRS4-AS1.

1. CPAT analyses of the protein-coding potential of DHRS4-AS1. B) The ORF of lncRNA DHRS4-AS1 in txCdsPredict.


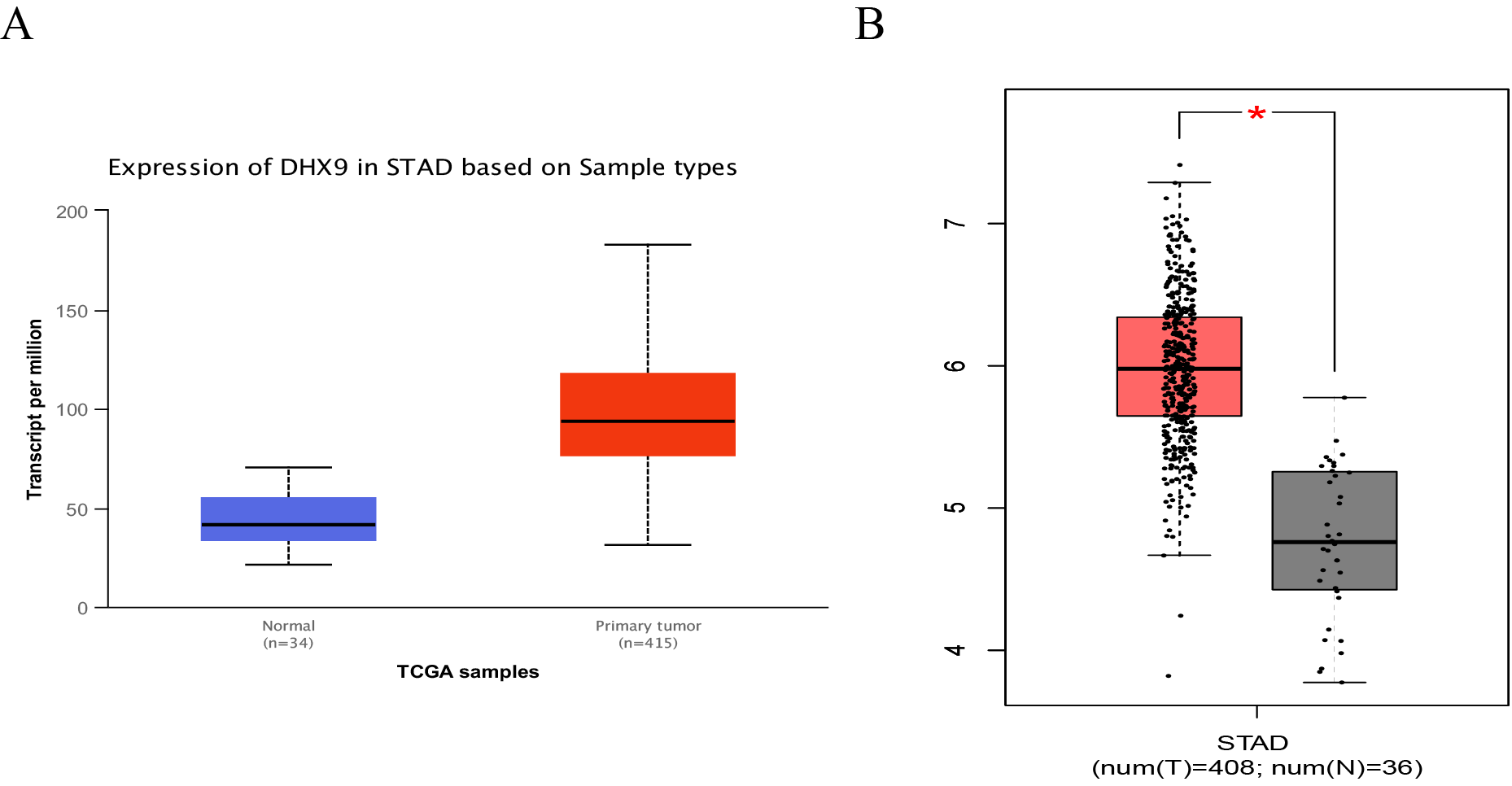


Fig. S2: The expression profile of DHX9 in TCGA and GEPIA databases.

1. DHX9 expression in gastric cancer was searched in the TCGA database. B）The expression of DHX9 in GEPIA database.

**Supplementary Table 1. Sequences of shRNAs and siRNAs**

| **Name** | **shRNA Sequence** |
| --- | --- |
| Sh-NC | CCGGTAGTCGCATACGGAACATTCGCTCGAGCGAATGTTCCGTATGCGACTATTTTTG |
| Sh-DHRS4-AS1 #1 | GAACTGTACTCTTACTCGAGTAAGAGTACAGTTCTGTCCTTTT |
| Sh-DHRS4-AS1 #2 | GCAGAGACCUUCCUGAAATTUUUCAGGAAGGUUCUCUGCTT |
| Sh-DHX9-NC | TTCTCCGAACGTGTCACGT |
| Sh-DHX9-1 | GGGCTATATCCATCGAAATTT |
| Sh-DHX9-2 | ACGACAATGGAAGCGGATATA |
| Si-ILF3 | CCUGUGUGAGAAAUCCAUU |

**Supplementary Table 2. Primers for RT-qPCR**

| **Name** | **Primer Sequence** |
| --- | --- |
| DHRS4-AS1-F | ATCTACCTTCCGCCTGACTGG |
| DHRS4-AS1-R | TCCAGAACTCGATGTGCTCCA |
| DHX9-F | GCCAATTTCTGGCCAAAGCA |
| DHX9-R | CGAGGCTCAATGGGGAGTTT |
| GAPDH-F | GGAAGCTTGCATCAATGGAAATC |
| GAPDH-R | TGATGACCCTTTTGGCTCCC |

**Supplementary Table 3. Antibodies used in the study**

| **Name** | **Company** | **Catalog Number** | **Antibody solubility** |
| --- | --- | --- | --- |
| Bcl-2 (Rabbit) | Abcam | Ab32124 | 1:1000 |
| Bax (Mouse) | Proteintech | 60267-1-Ig | 1:5000 |
| GAPDH (Mouse) | Proteintech | 60004-1-Ig | 1:5000 |
| DHX9 (Rabbit) | Proteintech | 67153-1-Ig | 1:5000 |
| DHX9 (Rabbit) | Abcam | Ab183731 | 1:5000 |
| Ubiquitin (Mouse) | CST | #3936 | 1:1000 |
| Normal Rabbit IgG (Mouse) | CST | #2729 | 1-5ug for IP |
| Mouse IgG (Mouse) | CST | #3420 | 1:20 for IP |
| MDM2 (Rabbit) | Abcam | Ab259265 | 1:1000 |
| MDM2 (Mouse) | Abcam | Ab16895 | 1:1000 |
| ILF3 (Rabbit) | Proteintech | 19887-1-AP | 1:5000 |
| Flag (Rat) | Abcam | Ab213519 | 1:1000 |
| Myc （Rabbit） | Abcam | Ab289980 | 1:5000 |
| NF-κB(P65) (Rabbit) | CST | #8242S | 1:1000 |
| p-P65 (Rabbit) | CST | #3033S | 1:1000 |
| IκBα (Mouse) | Proteintech | 10268-1-AP | 1:5000 |
| p-IκBα (Ser32) | CST | #2859S | 1:1000 |
| β-Actin | CST | #3700S | 1:1000 |

**Supplementary Table 4. Mass spectrometry of proteins pulled-down by lncRNA DHRS4-AS1 in AGS nucleus extracts**

| **No.** | **Protein names** | **Gene names** | **LFQ intensity Antisense (Nuclear protein)** | **LFQ intensity Sense (Nuclear protein)** |
| --- | --- | --- | --- | --- |
| 1 | Polyadenylate-binding protein 2 | PABPN2 | 0 | 61565000 |
| 2 | Polyadenylate-binding protein 1 | PABPC1 | 0 | 33842000 |
| 3 | GTP-binding nuclear protein Ran | RAN | 0 | 22228000 |
| **4** | **ATP-dependent RNA helicase A** | **DHX9** | **0** | **18028000** |
| 5 | **Interleukin enhancer-binding factor 3** | **ILF3** | **0** | **12526000** |
| 6 | Protein MAL2 | MAL2 | 0 | 11381000 |
| 7 | Heterogeneous nuclear ribonucleoprotein Q | SYNCRIP | 0 | 8458800 |
| 8 | Interleukin enhancer-binding factor 2 | ILF2 | 0 | 8107800 |
| 9 | AMP deaminase 2 | AMPD2 | 0 | 8026300 |
| 10 | Elongation factor 1-beta | EEF1B2 | 0 | 7901300 |

**Supplementary Table 5 The top 20 potential ubiquitin E3 ligase that target to DHX9**

| **E3** | **E3GENE** | **SUB** | **SUBGENE** | **HOMO** | **PFAM** | **GO** | **NET** | **MOTIF** | **SCORE** |
| --- | --- | --- | --- | --- | --- | --- | --- | --- | --- |
| **Q86TM6** | **SYVN1** | **Q08211** | **DHX9** | **1** | **1.73** | **1.25** | **1.77** | **6.61** | **0.803** |
| O60315 | ZEB2 | Q08211 | DHX9 | 1 | 3.18 | 2.88 | 1.77 | 1 | 0.77 |
| Q9C035 | TRIM5 | Q08211 | DHX9 | 1 | 3.17 | 3.77 | 1 | 1 | 0.746 |
| P22681 | CBL | Q08211 | DHX9 | 1 | 1 | 2.88 | 1.87 | 2.8 | 0.744 |
| Q9Y252 | RNF6 | Q08211 | DHX9 | 1 | 1.73 | 1 | 1 | 6.61 | 0.742 |
| **Q00987** | **MDM2** | **Q08211** | **DHX9** | **1** | **1** | **2.93** | **1.7** | **2.8** | **0.737** |
| P19474 | TRIM21 | Q08211 | DHX9 | 1 | 2.47 | 3.77 | 1 | 1 | 0.725 |
| Q86Y01 | DTX1 | Q08211 | DHX9 | 1 | 1.73 | 2.88 | 1.84 | 1 | 0.724 |
| Q14258 | TRIM25 | Q08211 | DHX9 | 1 | 3.17 | 1.25 | 1 | 2.12 | 0.716 |
| P14373 | TRIM27 | Q08211 | DHX9 | 1 | 2.47 | 1.78 | 1.69 | 1 | 0.705 |
| Q9HCE7 | SMURF1 | Q08211 | DHX9 | 1 | 1 | 4.05 | 1.69 | 1.06 | 0.703 |
| Q9UM11 | FZR1 | Q08211 | DHX9 | 1 | 1 | 1.78 | 1.77 | 2.12 | 0.695 |
| Q99973 | TEP1 | Q08211 | DHX9 | 1 | 1 | 3.77 | 1.77 | 1 | 0.695 |
| Q96Q27 | ASB2 | Q08211 | DHX9 | 1 | 1 | 1 | 1 | 6.61 | 0.694 |
| Q96PU5 | NEDD4L | Q08211 | DHX9 | 1 | 1 | 2.33 | 1.29 | 2.12 | 0.691 |
| Q9NZS9 | BFAR | Q08211 | DHX9 | 1 | 3.17 | 1.51 | 1.29 | 1 | 0.688 |
| Q9UNE7 | STUB1 | Q08211 | DHX9 | 1 | 1 | 3.77 | 1.87 | 1 | 0.676 |
| Q15751 | HERC1 | Q08211 | DHX9 | 1 | 2.47 | 1.25 | 1.77 | 1 | 0.676 |
| Q8TEQ6 | GEMIN5 | Q08211 | DHX9 | 1 | 1 | 3.77 | 1.7 | 1 | 0.676 |
